# Persistent myeloid cell reprogramming despite miltefosine treatment in leishmania-infected macaques

**DOI:** 10.1016/j.isci.2025.113900

**Published:** 2025-11-03

**Authors:** Morgane Picard, Steven Boutrais, Vasco Rodrigues, Yasmina Fortier, Chloé Borde, Calaiselvy Soundaramourty, Julien Clain, Charles Joly-Beauparlant, Gina Racine, Ouafa Zghidi-Abouzid, Arnaud Droit, Alain Pruvost, Maria Paola Costi, Ricardo Silvestre, Anabela Cordeiro da Silva, Jane MacDougall, Sónia André, Jérôme Estaquier

**Affiliations:** 1 INSERM-U1124, Université Paris Cité, Paris, France; 2 Centre de Recherche du CHU de Québec, Université Laval, Québec, QC, Canada; 3 Université Paris Saclay, CEA, INRAE, Département Médicaments et Technologies pour la santé, SPI, Gif-sur-Yvette, France; 4 Department of Life Sciences, University of Modena and Reggio Emilia, Modena, Italy; 5 Life and Health Sciences Research Institute (ICVS), School of Medicine, University of Minho, Braga, Portugal; 6 ICVS/3B’s-PT Government Associate Laboratory, Braga/Guimarães, Portugal; 7 i3S-Instituto de Investigação e Inovação em Saúde, Universidade do Porto, Porto, Portugal; 8 Parasite Disease Group, IBMC- Instituto de Biologia Molecular e Celular, Universidade do Porto, Porto, Portugal; 9 Departamento de Ciências Biológicas, Faculdade de Farmácia, Universidade do Porto, Porto, Portugal; 10 Photeomix, IP Research Consulting SAS, Noisy Le Grand, France

**Keywords:** Immunology, Parasitology

## Abstract

Visceral leishmaniasis (VL) is a neglected tropical disease caused by protozoan parasites. An inflammatory immune response, associated with tissue injury, occurs shortly after infection. Using a rhesus macaque model of VL, we evaluated the impact of miltefosine therapy administered during the acute phase of infection. Despite therapy, parasites persist in multiple tissues, including the spleen, bone marrow, peripheral, and mesenteric lymph nodes. Parasite burden inversely correlates with cellular miltefosine levels. Notably, *L. infantum* remains detectable three months post-treatment. Single-cell transcriptomic analysis reveals cellular heterogeneity and reprogramming of splenic myeloid cells post-treatment, including inflammatory macrophages, immature plasmacytoid dendritic cells, and type 2 dendritic cells (DCs). Flow cytometric sorting of splenic neutrophils, macrophages, and DCs confirms the presence of *L. infantum* post-treatment, highlighting the challenge of parasite clearance. Our findings reveal a disrupted innate immune landscape post-infection that persists after treatment, indicating myeloid cell reprogramming may sustain chronic infection and parasite persistence.

## Highlights

• Dissemination of *Leishmania infantum* in rhesus macaques
• Absence of parasite eradication despite early miltefosine treatment
• Parasites drive reprogramming of splenic myeloid cells toward an inflammatory state
• Sustained disruption of the innate immune landscape post-treatment

## Graphical abstract

**Figure:**
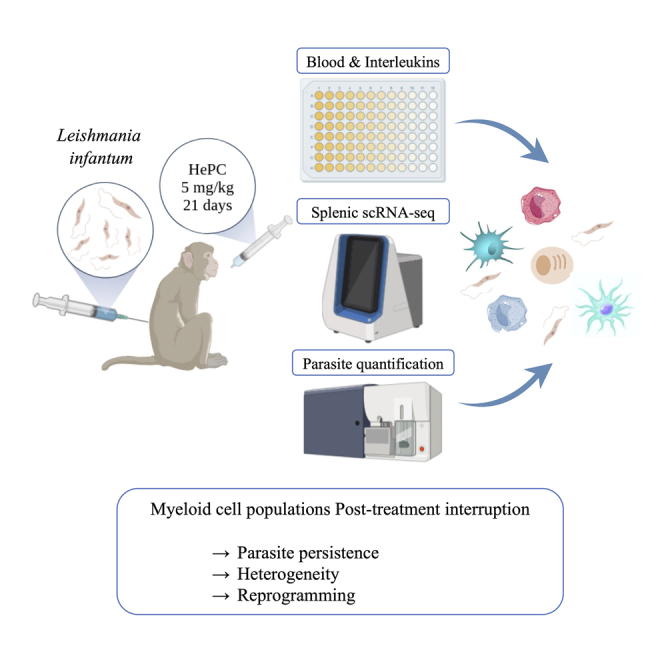

## Introduction

Leishmaniasis, a group of neglected tropical diseases (NTDs) caused by protozoan parasites of the genus *Leishmania*, manifests in three primary forms: cutaneous, mucocutaneous, and visceral leishmaniasis (VL). VL, the most severe form, causes nearly 20,000 deaths annually, predominantly in India, Sudan, Ethiopia, Somalia, and Brazil. In Southern Europe, approximately 700 new cases are reported annually,^1^^,^^2^^,^^3^ reflecting the geographic expansion of endemic areas driven by the presence of the sand fly vector.^4^ Several anti-leishmanial drugs are available, including amphotericin B, pentamidine, and miltefosine (hexadecylphosphocholine, *HePC)*.^5^ Despite their efficacy in reducing *Leishmania* burden, persistence and relapse have been reported.^6^^,^^7^

*Leishmania* predominantly infects myeloid cells, including monocytes, macrophages, dendritic cells, and neutrophils (PMNs).^8^ This is important because myeloid cells contribute to host defense and tissue homeostasis, in which tissue injury and inflammation influence myeloid cell fate. Macrophage polarization is classically divided into M1-like (pro-inflammatory) and M2-like (anti-inflammatory) phenotypes. In blood, monocytes are classified into classical (CD14^+^CD16^−^), intermediate (CD14^+^CD16^+^), and non-classical (CD14^−^CD16^+^) subsets, and they represent target cells.^9^ Furthermore, monocytes replenish tissue macrophages in the intestines, and the spleen serves as a reservoir of undifferentiated monocytes that are mobilized after injury.^10^^,^^11^ Once recruited to inflamed tissues, they can differentiate,^12^^,^^13^ giving rise to alternative macrophage subsets,^14^ tumor-associated macrophages (TAMs),^15^ or DC populations.^16^ Transcriptomic analyses have revealed during VL a shift toward a lymphoid inflammatory environment and a mixed M1/M2 signature in lymph nodes of dogs^17^ and spleen of hamsters.^18^^,^^19^ This is associated with accelerated splenic architecture breakdown,^20^ white pulp disorganization,^20^^,^^21^ and TNF-α production.^20^ Splenic aspirates from human VL individuals also indicate elevated TNF-α and interferon-gamma (IFN-γ) expression,^22^ although limited tissue access restricts human studies. In human blood, a type I IFN signature is observed in VL,^23^^,^^24^ however, mice lacking type I IFN signaling show early macrophage loss and splenic disorganization.^24^ It is important to note that inflammation and tissue injury occur early after infection, and TNF-α is detectable at day 3 post-infection.^20^ In mice, *L. infantum* induces inflammatory gene expression by day one, transitioning to regulatory immune signatures by day 10.^25^ Bioluminescence imaging shows that parasites colonize the spleen by day one post-infection^26^ and in rhesus macaques (RMs), which share physiological similarities with humans,^27^ early immune alterations, including hypergammaglobulinemia and splenic disorganization, were observed by day 11.^28^

Given the early immune changes and parasite establishment,^26^^,^^29^ we administered HePC to RMs daily from day one post-infection for 21 days, covering the acute phase. The goal was to assess parasite persistence and innate immune consequences after treatment interruption, rather than studying niche formation over time or traditional approaches that focus on treatment during the chronic phase. Our findings demonstrate that *L. infantum* persists in multiple tissues after HePC therapy interruption (HTi), with an inverse correlation between cellular drug concentration and parasite burden in RMs. Single-cell RNA sequencing of splenic cells reveals heterogeneity and reprogramming of myeloid cells, as well as an altered innate immune landscape that persists post-treatment. Collectively, these results advance our understanding of how *Leishmania* establishes and maintains infection within host tissues, even under early HePC therapy, and identify potential cellular targets to improve treatment outcomes for VL.

## Results

### HePC treatment in *L. infantum-*infected rhesus macaques

**Figure 1:**
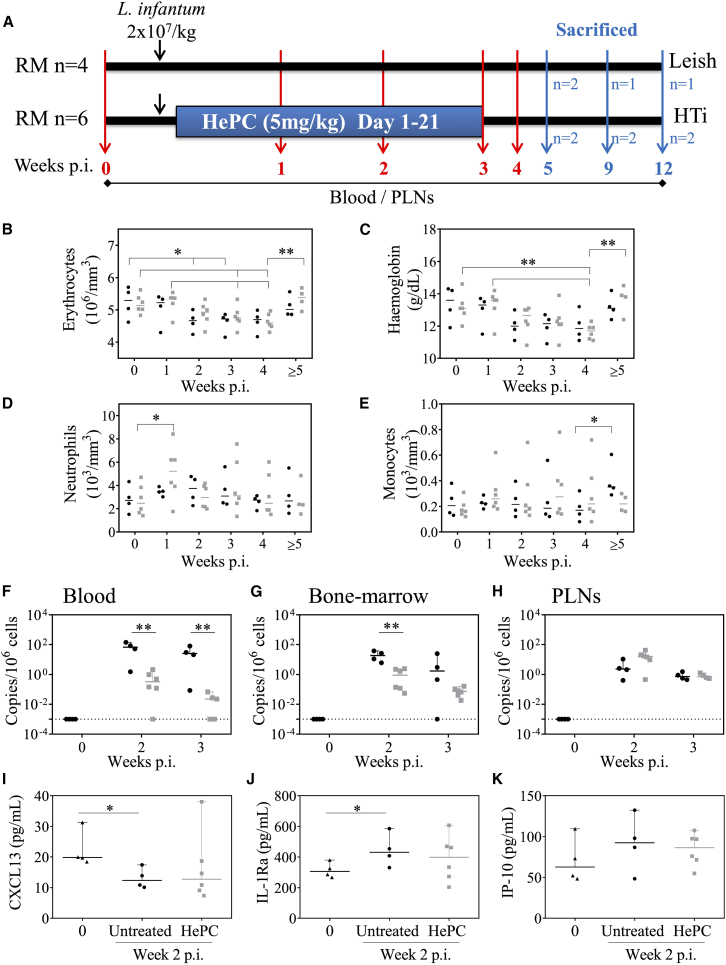
Impact of HePC therapy in *L. infantum*-infected RMs (A) Experimental scheme: Female RMs were inoculated intravenously with 2x10^7^*L. infantum* parasites/kg. At day 1 post-infection (p.i.), 6 RMs were treated for 21 days by nasogastric gavage with 5 mg/kg of HePC. Blood and PLNs were collected at different time-points p.i. (weeks 0, 1, 2, 3, 4, 5, 9, and 12). RMs were sacrificed at three time points (weeks 5, 9, and 12 p.i., corresponding to weeks 2, 4, and 9 post-HTi). (B-E) Blood samples were analyzed for (B) erythrocytes, (C) hemoglobin, (D) neutrophils, and (E) monocytes. (F-H) Parasite DNA was quantified by qPCR from (F) blood, (G) bone marrow, and (H) peripheral lymph nodes (PLNs) at weeks 2 and 3 p.i. (black circles: *L. infantum*-infected RMs; gray squares: *L. infantum*-infected RMs treated with HePC). (I–K) Levels of (I) CXCL13, (J) IL-1Ra, and (K) IP-10 were measured at week 2 p.i. Each point represents one individual RM. Statistical significance was assessed using the Mann-Whitney *U* test (∗, *p* < 0.05; ∗∗, *p* < 0.01).

RMs were intravenously infected with *L. infantum* promastigotes and subsequently treated with a daily oral dose of HePC (5 mg/kg) via gavage, starting on day 1 post-infection for 21 consecutive days, covering the acute phase (Figure 1A). This regimen mirrors the clinically approved dose used in humans.^30^^,^^31^ RMs were euthanized at weeks 5, 9, and 12 post-infection (2, 4, and 9 weeks post-HTi) to monitor *L. infantum* persistence in relation to HePC pharmacodynamics across tissues.

Following infection, animals developed transient anemia, characterized by reduced erythrocyte counts (Figure 1B) and lower blood hemoglobin levels (Figure 1C), as previously reported.^28^ HePC administration did not reverse this anemia (Figures 1B and 1C, gray squares). Early transient neutrophilia was observed in HePC-treated RMs but not in untreated controls (Figure 1D), whereas monocyte levels remained similar across the groups (Figure 1E).

Parasite burdens at weeks 2 and 3 post-infection were assessed by quantitative PCR (qPCR) targeting parasite kinetoplast DNA in blood, BM aspirates, and PLNs (Figures 1F–1H). By week 3, parasite loads dropped by 99% in HePC-treated RMs compared to controls in both blood (32.8 ± 34 vs*.* HePC, 0.02 ± 0.03 copies/10^6^ cells) and BM (7.1 ± 12 vs*.* HePC, 0.08 ± 0.05 copies/10^6^ cells) (Figures 1F and 1G). However, no significant reduction was observed in PLNs (0.9 ± 0.5 vs*.* HePC, 0.8 ± 0.3 copies/10^6^ cells, Figure 1H).

At week 2 post-infection, we also assessed by ELISA, CXCL13, a marker of germinal center activity,^32^ reported to be reduced in the spleen of dogs with canine leishmaniasis,^33^ and IL-1Ra, an anti-inflammatory mediator^34^ associated with pathogenesis.^35^ *L. infantum*-infected RMs showed decreased CXCL13 concentrations and increased IL-1Ra levels compared to uninfected RMs (Figures 1I and 1J), although substantial variation was observed under treatment. The IFN-inducible chemokine CXCL10/IP-10 tended to increase during acute infection in both treated and untreated *L. infantum*-infected RMs, but this change did not reach statistical significance due to inter-individual variability (Figure 1K). Overall, HePC administration during the acute phase of infection has a minor impact on parasite burden in PLNs and has a limited impact on hematological and inflammatory parameters.

### *L. infantum* detection despite HePC treatment

**Figure 2:**
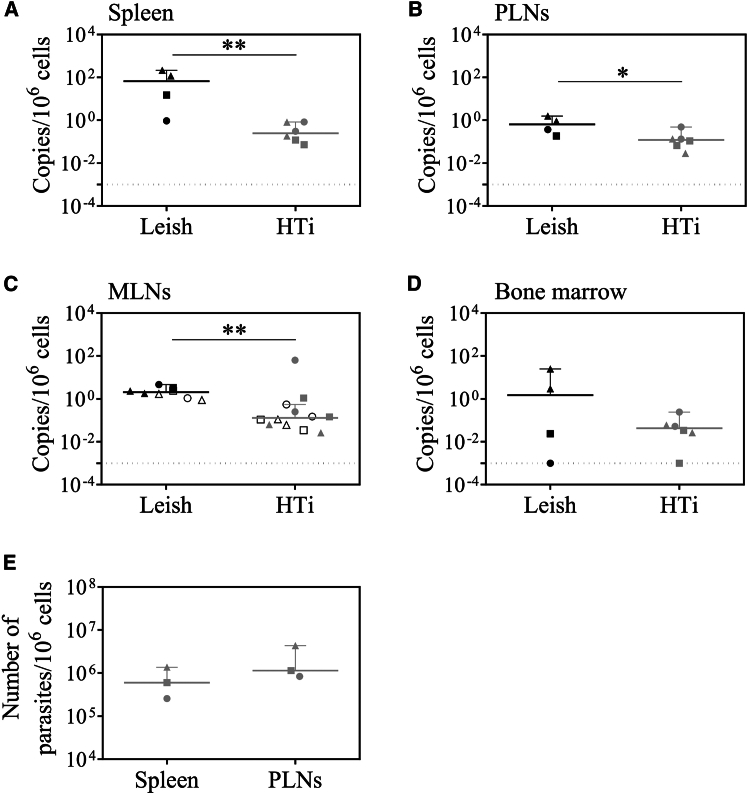
Parasite burden in *L. infantum-infected RMs* after HePC interruption (A–D) Parasite DNA was assessed by qPCR in RMs either infected with *L. infantum* parasites (Leish, *n* = 4) or treated with HePC (HTi, *n* = 6). Animals were sacrificed at different weeks post-infection (week 5, triangle symbol; week 9, square symbol; and week 12, circle symbol) in the (A) spleen, (B) PLNs, (C) MLNs (filled symbols are MLNs that drain the small intestine and open symbols are those that drain the large intestine), and (D) bone marrow of RMs. (E) Parasite rescue assay was assessed using the culture microtitration of splenic and PLNs cell suspensions from treated RMs sacrificed at weeks 5 (*n* = 1), 9 (*n* = 1) and 12 (*n* = 1) post-infection. Each data point represents one individual RM. Statistical significance was assessed using the Mann-Whitney *U* test (∗, *p* < 0.05; ∗∗, *p* < 0.01).

Since parasites persist in PLNs despite early HePC treatment, we quantified tissue parasite burden in RMs sacrificed at 2, 4, and 9 weeks post-HTi using qPCR. Parasites were quantified in the spleen, PLNs, BM aspirates, and MLNs, which drain the small and large intestines,^36^ a known anatomical site for parasite presence in dogs.^37^ In untreated RMs infected with *L. infantum*, the spleen was the most heavily parasitized organ (87.3 ± 100 copies/10^6^ cells), 50–100 times higher than in MLNs (2.3 ± 1.2 copies/10^6^ cells) or PLNs (0.75 ± 0.6 copies/10^6^ cells) (Figures 2A–2C). HePC-treated RMs exhibited a significantly lower parasite burden in the spleen and MLNs (Figures 2A and 2C), with a similar trend in PLN and BM (Figures 2B and 2D). Thus, the parasite burden for treated RMs after HTi was 0.4 ± 0.3 copies/10^6^ cells in the spleen, 5.5 ± 18.2 copies/10^6^ cells in MLNs, and 0.2 ± 0.1 copies/10^6^ cells in PLNs (Figures 2A–2C). Because qPCR may amplify DNA from non-viable parasites,^38^ we performed a parasite rescue assay^39^ to confirm the presence of viable parasites. Splenic and PLN cell suspensions of HTi RMs were serially diluted and incubated for 21 days to allow promastigote conversion and multiplication. Our data revealed an *in vitro* growth of *L. infantum* from both the spleen and PLNs of HTi RMs (Figure 2E). Therefore, despite early HePC treatment, viable parasites persist in various lymphoid organs, supporting *L. infantum* survival within host tissues.

### HePC pharmacodynamics

**Figure 3:**
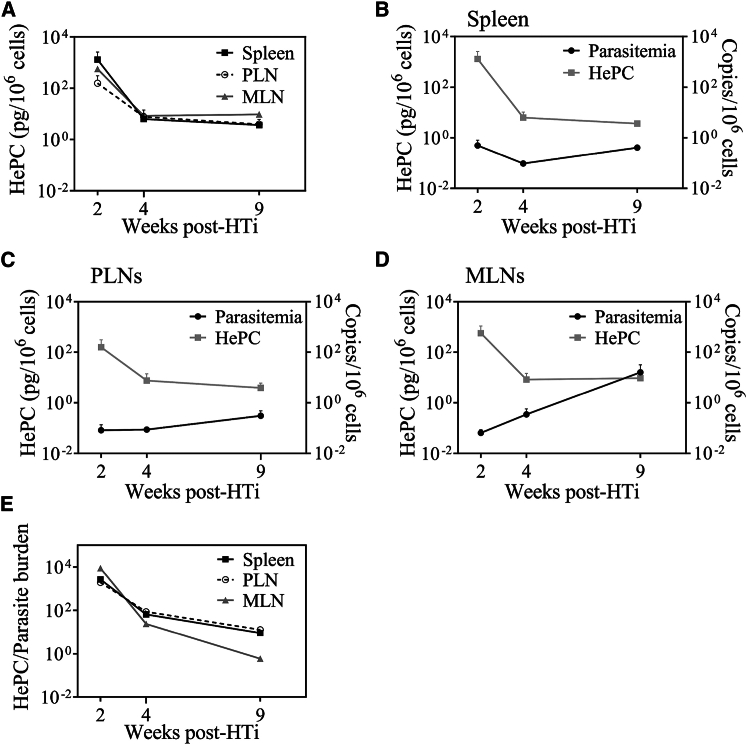
Pharmacodynamics of HePC in *L. infantum*-infected RM (A) HePC was quantified by UPLC-MS in splenic, PLNs, and MLNs cell pellets (10^6^ cells) at weeks 2 (*n* = 2), 4 (*n* = 2) and 9 (*n* = 2) post-HTi. (B–D) HePC quantification was plotted against the amount of parasitic DNA at the same time points for (B) Spleen, (C) PLN, and (D) MLN. (E) Ratio of HePC versus parasite quantification in the spleen, PLNs, and MLNs at weeks 2, 4, and 9 post-HTi. Each data point represents the mean ± SEM of two RMs.

Considering parasite persistence despite HePC administration, we quantified the remaining HePC levels in the spleen, PLN, and MLN cellular pellets at the time of sacrifice using UPLC-MS (Figure 3A). Two weeks after HTi, HePC levels remained elevated, declining but were still detectable at week 9 post-HTi (Figure 3A). At week 2 post-HTi, splenic HePC cellular levels (1308 ± 1824 pg/10^6^ cells) were twice those in MLNs (563 ± 769 pg/10^6^ cells) and 10-fold higher than in PLNs (156 ± 214 pg/10^6^ cells) (Figures 3B–3D). To evaluate the relationship between drug concentration and parasite control, we calculated the HePC-to-parasite burden ratio in each organ (Figure 3E). Despite lower HePC levels in PLNs and MLNs, the ratios were comparable to those in the spleen, suggesting similar parasite reduction across tissues at week 2 post-HTi (Figure 3E). However, at week 9 post-HTi, MLN displayed an increased parasite burden relative to earlier time points, suggesting a potential resurgence of parasitic replication in gut-associated lymphoid tissues (Figure 3D). Although HePC accumulation varied across lymphoid tissues, cellular HePC levels appeared insufficient to achieve complete parasite clearance, resulting in persistent *L. infantum* within these anatomical sites.

### Identification of myeloid cell populations in rhesus macaques using single-cell transcriptomic analysis

**Figure 4:**
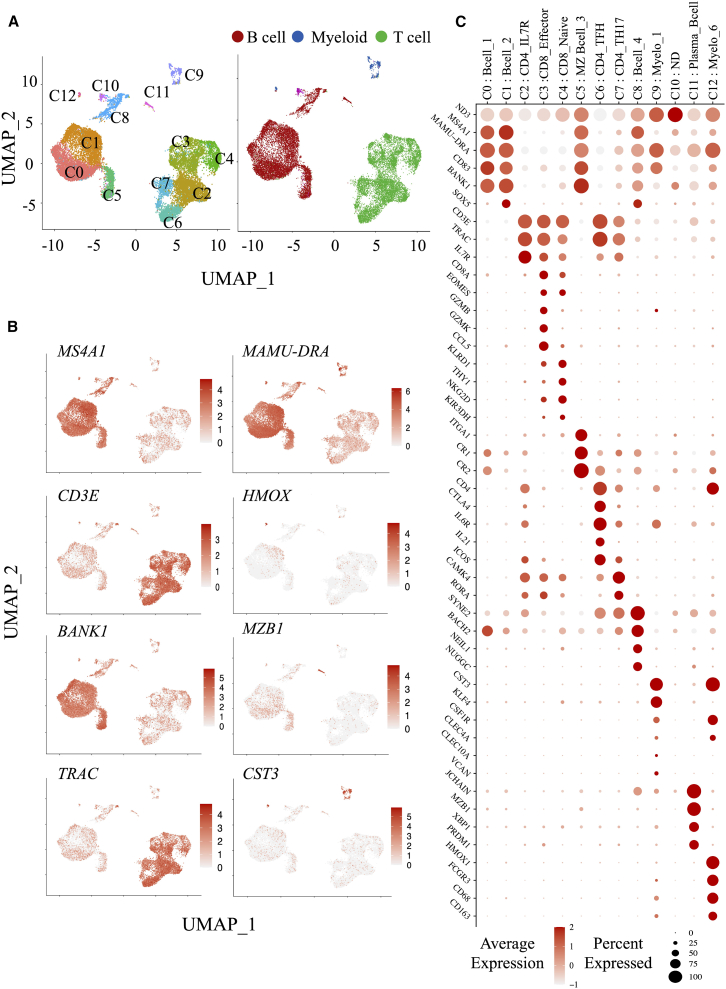
Single-cell transcriptome of spleen cells from RMs (A) By using a uniform manifold approximation and projection (UMAP) unsupervised method, we classified immune cells from spleen samples into 13 clusters (C0 to C12). Each cluster is shown with different colors and can be divided into three main populations (B cells, myeloid cells, T cells). (B) *MS4A1*, *MAMU-DRA*, *BANK1,* and *MZB1* define B cells, *CD3E* and *TRAC* define T cells, and *MAMU-DRA*, *HMOX* and *CST3* define myeloid cells. Scale of relative gene expression is shown. (C) Heatmap highlighting genes for cluster identification. The size of the dot represents the percentage of cells expressing the selected gene in each cluster and its average expression (see also Figure S1).

Due to the lower abundance of myeloid cells in LNs, we focused on the spleen, where these cells are more prevalent and the *L. infantum* niche are established in RMs. Single-cell RNA sequencing (scRNA) was performed to assess splenic cellular heterogeneity in naive, *L. infantum*-infected at week 5 post-infection, and HTi RMs at weeks 5 and 9 post-infection (corresponding to weeks 2 and 4 post-HTi). This approach enables high-resolution profiling of gene expression to identify immune cell subsets.^40^ Splenic cells were isolated using Ficoll-Paque density gradient centrifugation to enrich viable cells, although this excludes neutrophils. CD45^+^ cells were stained and processed using the BD Rhapsody single-cell analysis system. Samples from all groups were multiplexed and processed simultaneously to minimize analytical bias. We loaded 15,000 tagged cells, achieving over 95% efficiency in capturing a single viable cell per bead, as confirmed by quality controls. Two independent experiments were performed and combined. After quality control and sample demultiplexing, we successfully obtained a total of 100,460,858 counts across 19,066 cells with a median of 5,200 reads per cell. These included 3,201 cells from naive, 4,716 cells from *L. infantum-*infected, and 9,300 cells from HTi RMs (weeks 2 and 4 post-HTi). Unsupervised clustering using Seurat identified 13 clusters (C0 to C12). The top 10 differentially expressed genes (DEGs) are shown in the heatmap (Figure S1). The uniform manifold approximation and projection (UMAP) visualization of splenic immune cell populations in RMs is shown in Figure 4A. Based on well-known genes that are specifically assigned to T and B cell identities, such as *MS4A1, BANK1, MAMU-DRA, MZB1, CD3E,* and *TRAC*, we identified the main lymphoid populations (Figure 4B). The prominent gene signatures for each cluster are displayed in the supervised heatmap with the relative average gene expression against the percentage of expressed cells (Figure 4C). B cells (*MS4A1* and *BANK1*) were enriched in clusters 0, 1, 5, 8, and 11. While complement receptors, *CR2* (CD21) and *CR1* (CD35), identify marginal zone (MZ) B cells in cluster 5, the expression of *JCHAIN*, *MZB1,* and *XBP1* transcripts identifies plasma B cells in cluster 11. The expressions of *CD3E* and *TRAC,* as well as *CD8A* (including naive and effector cell subsets) and *CD4* transcripts (including CD4^+^IL7R^+^, Th17, and T follicular helper (Tfh) cell subsets), define T cell subsets in clusters 2, 3, 4, 6, and 7. Thus, Tfh cells are identified by *CTLA4*, *IL6R, IL21,* and *ICOS* transcripts, while *RORA* and *CAMK4* are associated with Th17. In contrast to their protein-level surface expression, *CD4* and *CD8* transcript levels are low, consistent with a recent report.^41^ *MAMU-DRA*, which is highly expressed in B cell clusters, is also prominently expressed in clusters 9 and 12. Myeloid cells were identified by *CST3*, *CSF1R*, *CLEC4A*, *CLEC10A*, *VCAN*, *HMOX1*, *FCGR3*, *CD68,* and *CD163* (Figures 4B and 4C), which were absent in cluster 10, leaving its identity undefined. Altogether, scRNA-seq provides a high-resolution mapping of splenic immune populations and reveals distinct myeloid clusters in *L. infantum* RMs.

### Heterogeneity of splenic myeloid cell populations

**Figure 5:**
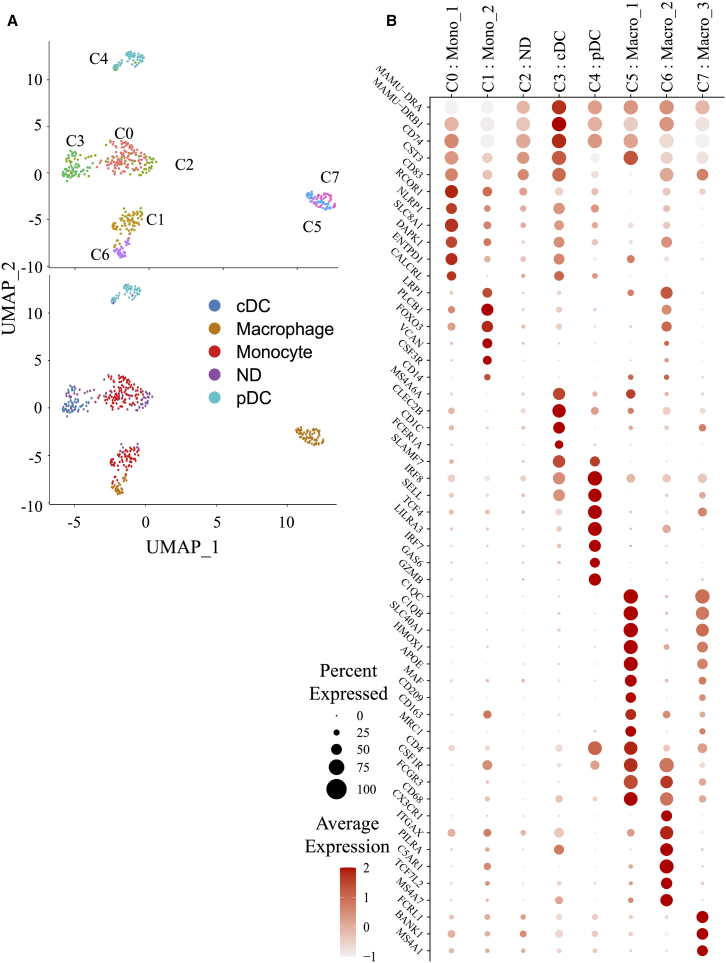
Single-cell and clustering of myeloid cells (A) UMAP projection of clusters 9 and 12 identified in Figure 4. The 8 clusters are indicated by different colors and correspond to cDC (blue), macrophage (brown), monocyte (red), pDC (light blue), and not defined (ND, purple). (B) Supervised heatmap highlighting genes of interest for myeloid clusters. The size of the dot represents the percentage of cells expressing the selected gene in each cluster and its average expression (see also Figure S2; Tables S2, S3, S4, S5, S6, S7, S8, S9, S10, S11, and S12).

After defining splenic myeloid cell clusters, we reran clusters 9 and 12 to define myeloid cell subsets in RMs. Seurat clustering analysis revealed eight distinct populations defined by their DEG profiles (Figures 5 and S2; Tables S1, S2, S3, S4, S5, S6, S7, S8, S9, S10, S11, S12, and S13). UMAP visualization of myeloid cell clusters and DEG heatmap are shown in Figure 5. Cluster 0 was enriched with 169 DEGs (FDR with ≥0.05 and log_2_FC > 1), including high expression of *RCOR1* (REST corepressor 1), *SCL8A1*, also known as *NCX1* (Sodium-calcium exchanger 1), *NLRP1* (NLR family pyrin domain containing 1), *DAPK1* (death associated protein kinase 1) and *ENTPD1* (CD39) (Figure 5B; Table S1). According to the Human Protein Atlas, these genes are associated with monocytes.^42^ Among the 35 specific genes in cluster 1, *VCAN*, *CSF3R,* and *CD14* transcripts are related to monocytes (Figure 5B; Table S1). In cluster 3 (287 DEGs) (Table S1), the gene profile corresponds to a DC signature (*MAMU-DRA, MAMU-DRB1, MS4A6A* (CD20L3)*, CLEC2B, CD1C,* and *FCER1A*) (Figure 5B). Enrichment of transcripts encoding for *IRF8*, *SELL* (CD62L)*,* leukocyte immunoglobulin like receptor A3 (*LILRA3* ), and *GZMB* (Figure 5B; Table S1) indicates plasmacytoid dendritic cells (pDCs) in cluster 4 (332 DEGs). Cluster 5 comprises 141 DEGs enriched in *C1QC, C1QB, SLC40A1, HMOX1*, *APOE,* and *MAF*, characterizing M1 macrophages, while *CD163* and *MRC1* (CD206) transcripts are reminiscent to the M2 profile (Figure 5B; Table S1).^43^ A similar M1 signature (*C1QB, C1QC, SLC40A1*) is observed in cluster 7 (95 DEGs), along with an enrichment in B cell-associated transcripts such as *FCRL1, MS4A1* (CD20)*, CR2* (CD21)*, CD19,* and *IGHM* (Figure 5B; Table S1). This gene profile characterizes tingible body macrophages, which are involved in clearing dying cells within splenic B cell follicles.^44^ In addition to express *CSFR1, FCGR3* (CD16) and *CD68*, shared with cells in cluster 5, macrophages in cluster 6 (563 DEGs) express *CX3CR1* (fractalkine receptor), *ITGAX* (CD11c)*, PILRA, C5AR1,* and *TCF7L2,* which are associated with M2 polarization^45^ (Figure 5B; Table S1). Unfortunately, cluster 2 could not be clearly defined based on its gene expression profile. Therefore, scRNA-seq allows us to create a detailed map of myeloid cell subsets and reveals notable heterogeneity within the spleen of RMs.

### Redistribution of splenic myeloid cell subsets in *L. infantum*-infected that persists despite HePC therapy in rhesus macaques and HePC therapy interruption rhesus macaques

We compared the distribution of myeloid cell subsets in *Leishmania*-infected and HTi RMs to those in naive RM cells (Figure S3). UMAP projections for naive (Figure S3A), *Leishmania*-infected (Figure S3B), and HTi RMs (Figure S3C) illustrate changes in cluster composition and proportions (Figures S3D–S3F). The proportion of monocytes in cluster 0 decreased from 35.4% in naive RMs to 19.1% in *Leishmania*-infected and 18.3% in HTi RMs. In contrast, monocytes in cluster 1 increased from 7.6% in naive RMs to 16.2% in *Leishmania*-infected and 20.4% in HTi. Moreover, our results revealed higher proportions of pDC (cluster 4) in *L. infantum*-infected and HTi (20.6% and 14.2%, respectively) than in naive RMs (3.5%). The high proportion of these two myeloid cell populations in HTi RMs indicates that there are persistent innate immune alterations despite the treatment administered during the acute phase of infection. Comparing macrophage proportions in clusters 5 and 7 between naive RMs (14.6% and 6.9%, respectively, Figure S3D) and HTi RMs (6.3% and 8.3%, Figure S3F), we observed a notable reduction specifically in cluster 5. These two populations are rarely observed in *L. infantum*-infected RM. A major observation was the proportion of macrophages in cluster 6 that predominated in *L. infantum*-infected (22.1%, Figure S3E) and HTi RMs (7.9%, Figure S3F). These findings indicate a modification in the distribution of myeloid subsets during infection and post-HePC treatment, reflecting sustained immune remodeling and likely contributing to parasite persistence.

### Persistent reprogramming of monocytes despite HePC treatment

**Figure 6:**
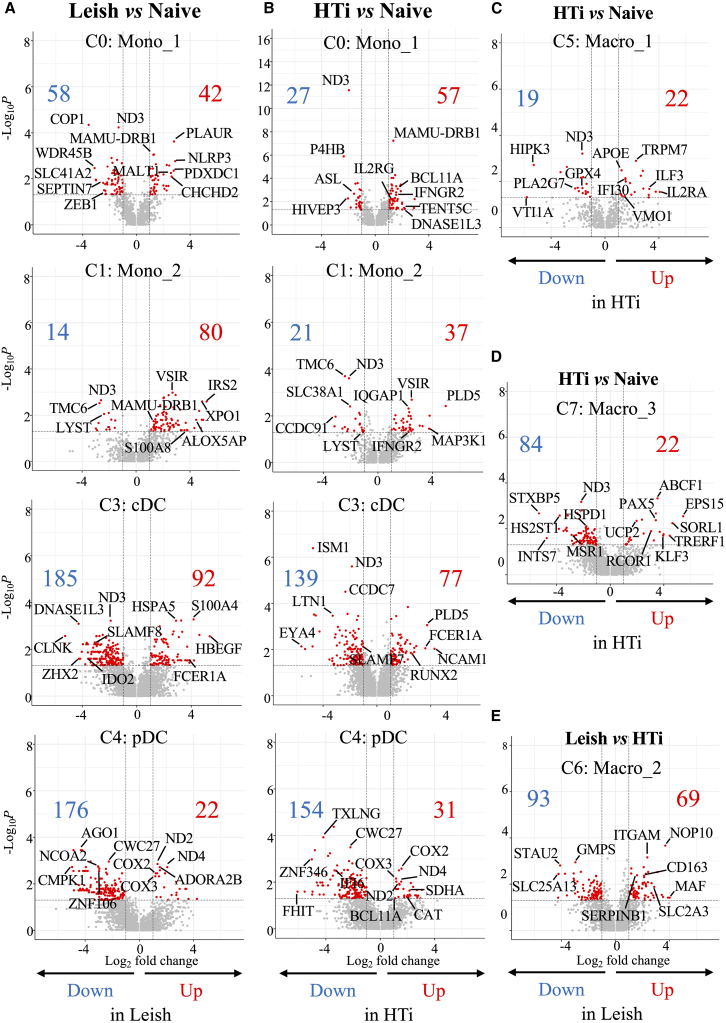
Differential expression gene (DEG) analysis for myeloid cells Volcano plot of differentially expressed genes between (A) *L. infantum*-infected (*Leish*) or (B) HTi RMs compared to naive RM (see Tables S2, S3, S4, S5, S6, S7, S8, and S9). The volcano plots illustrate the differences in the gene expression of cells in clusters 0 (mono_1), 1 (mono_2), 3 (cDC), and 4 (pDC) but also (C) cluster 5 and (D) cluster 7 for HTi versus naive RMs, and (E) cluster 6 for *L. infantum*-infected RMs compared to HTi. Red arrows and numbers indicate up-regulated transcripts, and blue arrows and numbers indicate down-regulated genes in specific RMs group. X axis displays the log2 fold change values, and the Y axis represents the Log_10_*p* values (see also Figures S4–S8).

To evaluate myeloid cell reprogramming in response to *L. infantum* infection and subsequent HePC treatment, DEG profiles were analyzed in specific myeloid cell populations, comparing *L. infantum*-infected and HTi to naive RMs (Figure 6; Tables S2, S3, S4, S5, S6, S7, S8, S9, S10, S11, and S12). Only genes with a 2-fold increase and at least two reads were considered, increasing the stringency of gene selection.

Monocytes (cluster 0) from *L. infantum*-infected exhibited enrichment in transcripts related to immune activation (e.g., *MAMU-DRB1*, *NLRP1*, *NLRP3*, *MALT1,* and *PLAUR*) and metabolic regulation, including *ATP11A* (flippase complex member), *ATP2B1* (plasma membrane calcium-transporting ATPase 1)*,* inositol polyphosphate-5-phosphatase (*INPP5F*) and protein phosphatase Mg^2+^/Mn^2+^ dependent 1E (*PPM1E*) (Figure S4; Table S2). Whereas HTi RMs showed partial overlap with this profile, with the persistent upregulation of *MAMU-DRB1* and *MALT1,* we noticed specific expression of interferon gamma receptor 2 (*IFNGR2* ), interleukin-2 receptor subunit gamma (*IL2RG*), lysozyme (*LYZ*), and *BCL11A*. Moreover, genes that regulate lipid metabolism and catalytic processes exhibited higher expression in HTi compared to naive RMs. These gene profiles show the reprogramming of myeloid cells toward inflamed monocytes that persist after HePC interruption (Figure S4C; Table S3).

Down-regulated genes in monocytes from *L. infantum*-infected (58 genes), and HTi (27 genes) compared to naive RMs (Figures 6A and 6B; Tables S2 and S3), were primarily linked to nuclear function (*BHLHE40*, *MAFF*, *RUNX1*), zinc finger factors (*ZBED1*, *ZBTB20*, *ZEB1*, *ZNF451* and *HIVEP1*), and mitochondrial metabolism, including acyloxyacyl hydrolase (*AOAH*), NADH dehydrogenase subunit 3 (*MT-ND3* ), induced myeloid leukemia cell differentiation protein Mcl-1 (*MCL1*), a pivot member of the anti-apoptotic BCL-2 family proteins,^46^ *TANK* (TRAF family member-associated NF-κB Activator) and Niban apoptosis regulator 1 (*NIBAN1)* , indicating compromised mitochondrial function and survival expression of cDC1-associated genes (*BBX*, *ZEB1,* and *ZNF451*), suggesting that monocyte-to-DC may be impaired after infection, which persists after HePC treatment.

Monocytes (*VCAN*^+^, cluster 1) from *L. infantum*-infected compared to naive RMs up-regulated 80 genes (Figures 6A; Table S4), many associated with immune activation and inflammation, including *MAMU-DRA*, *MAMU-DRB1, NLRP1, CD86, ENTPD1* (CD39), *S100A4/8, RIPK2* and *MSR1* (macrophage scavenger receptor 1) (Figure S5A; Table S4). Several of these, notably *MAMU-DRB1*, *NLRP1,* and *VSIR (*V-Set immunoregulatory receptor), remain elevated in HTi RMs, suggesting persistent inflammation (Table S5). Genes involved in cell migration (*MYH9*, *MYL6*, *RAB1A*, *RAB31*, *RHOG*, *IQGAP1*, and *VIM*) and catabolic activity, such as *ALOX5AP* (arachidonate 5-lipoxygenase activating protein), *ALDOA* (aldolase, fructose-bisphosphate A), *HSPA5*, *USP8*, and *SLCO3A1,* were also enriched in *L. infantum*-infected RM cells (Figure S5C; Table S4). Ribosomal and metabolic transcripts were upregulated, reflecting increased protein synthesis and metabolic turnover (Figure S5A). The continued expression of *IFNGR2* highlights ongoing IFN-γ signaling and monocyte activation in both infected and treated RMs (Figure S5C; Table S5). Down-regulated genes were fewer (14 in infected and 21 in HTi RMs) and included regulators of enzyme activity, cellular transport, and organelle function (*AKT3, LYST, SCAPER*), along with *MT-ND3*, reinforcing evidence of mitochondrial dysfunction^46^ (Figure S5; Tables S4 and S5). Overall, these findings confirm the reprogramming of monocytes into an activated, infiltrating myeloid cell population that persists after HePC interruption.

### Polarization of conventional dendritic cells toward type 2 despite HePC treatment

We extended the analysis to conventional DCs (cluster 3), revealing substantial transcriptional reprogramming in both *L. infantum*-infected (92 up- and 185 down-regulated genes) and HTi RMs (77 up- and 139 down-regulated genes) (Figures 6 and S6; Tables S6 and S7). They express high levels of *FLT3* transcripts,^47^ displaying a conventional type 2 (cDC2) profile. While cDC2 markers (*FCER1A* and *KLF4*) were enriched in infected cells,^48^ key cDC1-related genes (*CLEC9A*, *IDO2*, *SLAMF7*, *SLAMF8*, *RELB*, *SERPINB9*, *ZNF366, DNASE1L3*, *FUCA1,* and *MLEC*),^49^ and monocyte-associated genes (*CD84*, *TNFAIP3*, *CTBS*, *CFLAR*, *DUSP18*, *KDM4C*, and *ZNFX1*) were down-regulated, patterns largely retained post-treatment (Figure S6C; Tables S6 and S7). This suggests persistent impairment of cDC1 identity. Down-regulated genes also included *NFKB1*, *RELB*, *IRF2, TNFAIP3* (A20, a regulator of NF-kB signaling),^50^ as well as genes involved in TBK1 activation (*TBC1D8* and *ZNF268*), which are crucial for innate immune regulation (Table S6). We also observed a lower expression of mitochondrial/OXPHOS components (*MFN1*, *NDUFB4*, *UQCRQ*, *ND3,* and *UCP2*), supporting a metabolic shift toward fatty acid oxidation, evidenced by increased *ACOX1*, *HACD4,* and *MGLL* expression (Table S6). Higher expression of *RUNX2, RUNX3*,^51^^,^^52^ *FOXO1,* and *ATF3*^53^^,^^54^ in HTi DCs further reinforces the link to TGF-β-mediated tolerogenic programming associated with *NR4A1/A2* (Nur77 and Nurr1).^55^ Overall, scRNA-seq revealed a sustained cDC2-biased transcriptional profile following infection, unaffected by HePC treatment.

### Plasmacytoid dendritic cell alterations reflect mitochondrial and autophagy stress

Transcriptomic analysis of pDCs (cluster 4) revealed major gene expression changes in *L. infantum*-infected (22 up- and 176 down-regulated genes) and in HTi RMs (31 up- and 154 down-regulated genes) compared to naive RM (Figures 6A, 6B, and S7; Tables S8 and S9). Enriched transcripts included mitochondrial DNA genes encoding for cytochrome *c* oxidase (*MT-COX*), NADH dehydrogenase (*MT-ND*), and ATP synthase (*MT-ATP6*), suggesting mtDNA replication. The upregulation of *ADORA2B* (hypoxia/adenosine sensing),^56^ *BMT2* (S-adenosylmethionine (SAM) sensor),^57^ *EIF2AK4* (or GCN2), a sensor of amino acid starvation,^58^ *GADD45B* (cell-cycle arrest and apoptosis), and *JUN* (stress response) further indicated cellular stress. HTi pDCs also showed elevated *RUBCNL* (autophagy),^59^ *BCL11A* (enhances Bcl-2 and pDC development),^60^ and *PRXL2A* (redox regulation),^61^ reinforcing the notion of autophagy activation and mitochondrial stress in response to infection (Figures S7A and S7B; Table S9).

Concurrently, a broad down-regulation of genes linked to mitochondrial function (*AKR7A2, ATP5F1A, CS, OXR1, TUFM*, and *UQCRC2*), lysosomal/endosomal pathways (*AP2M1, ARPC2, GAS7, LAPTM4A, LAPTM5, RAB21, RER1, ROCK2, VPS35L*), and catabolic processes (*PIAS1, PSMD14, PSMD7, PSME1, UBE4A, UBE2E1,* and *UCHL5*) was observed (Figures 6A, 6B, and S7; Tables S8 and S9). Transcripts involved in RNA metabolism and gene expression, including *NAF1* (ribosome biogenesis), *NSD1* (histone methyltransferase), *POLDIP3/D4* (DNA polymerase delta complex), and *TAF1/7* (TATA-box binding protein associated factors) were also suppressed, suggesting impaired transcriptional regulation. Notably, multiple zinc finger proteins (*ZNF106, ZNF124,* and *ZNF346*) and regulators of pDC function within the TGF-β and Notch pathways (*MBP, NOTCH2, TGFBR1,* and *TAB2*) were also diminished^62^ (Figure S7C; Tables S8 and S9). Collectively, these data point to a sustained dysfunctional profile in pDCs post-HePC, characterized by metabolic and organelle dysregulation.

### Persistent macrophage reprogramming marks incomplete visceral leishmaniasis resolution

In macrophages (*C1QB + C1QC + SLC40A1+,* cluster 5), 22 genes were upregulated in HTi compared to naive RMs (Figure 6C; Table S10). Gene enrichment suggests a tumor-associated macrophage (TAM) profile, consistent with the M1/M2 classifier.^63^ These genes included *APOE*,^64^ *ILF3* (cholesterol homeostasis),^65^ *IFI30* (lysosomal thiol reductase), and *IL2RA***.** In contrast, down-regulated genes were primarily associated with metabolism and ferroptosis. These include *FABP4* (triglyceride synthesis),^66^ *GPX4* (protects against oxidized lipids),^67^ *MT-ND3*, *NDUFA1, CPVL* (carboxypeptidase vitellogenic like), *GLUL* (glutamine synthetase), *PLA2G7* (phospholipase A2 Group VII), and membrane spanning 4-domains A6A (*MS4A6A*) (Figure S8A; Table S10). This is notable since, along with *MS4A6A*^68^*,* inhibition of PLA2G7 reverses the immunosuppressive function of macrophages,^69^ regulating M1/M2 polarization (Figure S8A; Table S10). Hence, despite the administration of HePC, macrophages are reprogrammed into a metabolic status more permissive to ferroptosis.

In tingible body macrophages (cluster 7), 22 genes were up- and 84 down-regulated in HTi RMs compared to naive animals (Figure 6D). Enriched genes included transcriptional repressors known to inhibit myeloid cell function, such as *KLF3* (NF-κB regulator),^70^ *PAX5* (CSF1R repressor),^71^ *RCOR1* (REST corepressor 1),^72^ *TRERF1* (activates p21 and p27 promoters)^73^ and *PPP1R18* (NFATc1 suppressor).^74^ Increased expression of motility-related genes such as regulates actin cytoskeleton (*AHNAK*),^75^ actin depolymerizing factor (*DSTN*), *CEMIP2*, *NCKAP1L* (component of the actin-regulatory WAVE complex),^76^ and sortilin-related receptor (*SORL1*)^77^ strongly suggests dynamic cytoskeletal remodeling.

In contrast, the down-regulated genes were primarily linked to immune effector function and organelle integrity. These include cell surface molecules, such as *CFP* (properdin), *CR2*, *IFNGR1*, *MSR1* (macrophage scavenger receptor),^78^ *OSBPL2* (an LDL-derived cholesterol transporter), along with immunoregulatory receptors prostaglandin E2 receptor (*PTGER4*) and *PGRMC2* (progesterone receptor)^79^ (Figure S8B; Table S11). This is associated with reduced levels of *TBK1,* reflecting dampened innate signaling^80^ (Figure S8B; Table S11) but also genes related to mitochondrial functions (*NAMPT*, *OPA1*, *MT-ND3* and *HSPD1* (HSP60),^46^ apoptosis (*BCL2L11* or BIM and *BCLAF1*), and ER morphogenesis (*RTN4*).^81^ Down-regulation of E3 ubiquitin ligases (e.g., *PELI1, UBR5, ARIH1, CBLB, HERC3*) further supports impaired cellular maintenance (Table S11). Overall, these results indicate that despite HePC treatment, tingible body macrophages are reprogrammed into a dysfunctional state marked by impaired organelle architecture and reduced immune competence, potentially increasing susceptibility to cell death through autocrine regulation.

A distinct M2 macrophage population (cluster 6), linked to tissue repair and inflammation,^82^ was identified in *L. infantum*-infected and HTi RMs (Figure S3). Relative to HTi, infected RMs exhibit 69 up-regulated genes (Figure 6E), including *CD163*, *CXCR4*, *IL4R*, and *ITGAM* (CD11b) (Figure S8C; Table S12) along with mitochondrial transcripts (*ASCL4*, *COX17*, *MTCH2*, *ENO1*, *UQCRB,* and *VDAC1*), and redox-related genes (*GLRX*, Glutaredoxin, and *PRDX5*, Peroxiredoxin 5) (Figure S8C; Table S12). Genes involved in cell cycle control (*CCND3*, *CCNG2*, *CDC5L,* and *PCNA*) were also elevated, indicating active macrophage proliferation following *Leishmania* infection.

In contrast, macrophages from HTi RMs exhibited up-regulation of genes related to protein quality control and autophagy, including *ERLEC1* (XTP3-B, ER-associated degradation), *TAX1BP1* (autophagic receptor for aggresome clearance),^83^ *OSBPL8* (transport of phosphatidylserine from ER)^84^ and two autophagic protein kinases, *ULK2*^85^ and *DAPK1*.^86^ Ubiquitination-related transcripts were also enriched: *COP1* (targets C/EBPα and regulates myeloid differentiation), *PIAS1* (regulates STAT1), and *TRIM8* (promotes TAK1 ubiquitination) (Figure S8C; Table S12). Notably, *MDM2*, a p53-regulator, and mitochondrial translation initiation factor IF-2 (*MTIF2* ), which stabilizes mitochondrial mRNA^87^ were elevated along with *MT-ND5* and *SLC25A13* (an aspartate/glutamate carrier), indicating mitochondrial reprogramming. Additional markers of metabolic adaptation and inflammation included *POU2F2*,^88^ *KLF3/7/12*,^89^ *SLC44A2*,^90^ and *NLRP1*,^91^ suggesting a shift toward aerobic glycolysis and a pro-inflammatory state in HTi macrophages (Figure S8C; Table S12). These findings support divergent fates: infected RMs exhibit proliferating, mitochondria-active M2 macrophages, while HTi RMs show a catabolic, inflammatory profile with metabolic rewiring.

### Identification of infected myeloid cells post-HePC treatment

**Figure 7:**
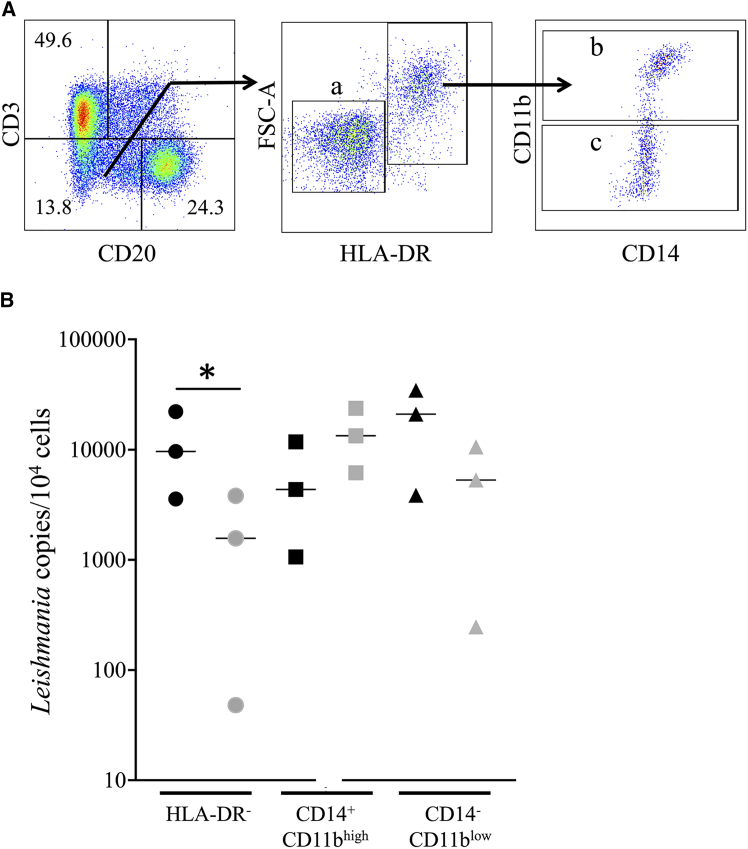
*L. infantum*-infected cells in the spleen Splenic cells of *L. infantum*-infected RM and HTi were sorted by flow cytometry. (A) Gating strategy was used to sort HLA-DR^-^ (a), HLA-DR^+^CD14^+^CD11b^high^ (b), and HLA-DR^+^CD14^−^CD11b^low^ (c) cell subsets, excluding lymphoid T cells (CD3^+^) and B cells (CD20^+^). (B) Parasite DNA was assessed by qPCR, and results are expressed as copy number per number of cells among infected (black symbols) or HePC-treated RMs (HTi, gray symbols). Each symbol represents the mean of three independent experiments performed in duplicate for each RM. The bars represent the median of 3 RMs. Statistical significance was assessed using the Mann-Whitney *U* test (∗, *p* < 0.05).

Dual alignment to the *L. donovani g*enome in an infected mouse was recently reported.^92^ In our study, 61,484 reads aligned with the *L. infantum* genome; however, over 99% of these mapped to intergenic or intronic regions and to the genome of the naive RM (Table S13). All *Leishmania*-mapped reads were heavily clipped, suggesting non-specific background signals. Thus, genome alignment did not reliably identify infected cells. To overcome this limitation, we used the flow cytometry sorting of splenic cells from both HTi RMs and *L. infantum*-infected RMs. Total splenic cells, including neutrophils, were stained with specific antibodies, bypassing Ficoll-Paque isolation. Splenic myeloid cell subsets were identified by CD11b and CD14 expression. After excluding lymphoid cells (T cells, CD3^+^CD20^−^ and B cells, CD3^−^CD20^+^), monocyte/macrophage (CD11b^high^ and CD14^+^) and dendritic cell subsets (CD11b^low^CD14^-^) were sorted from the HLA-DR^+^ population, while neutrophils were mostly present in the HLA-DR^-^ population (Figure 7A). The presence of parasites was assessed by qPCR in sorted populations from both *L. infantum*-infected (*n* = 3) and HTi (*n* = 3) RMs. Results, presented as the mean of three experiments performed in duplicate, confirmed parasite DNA in CD11b^high^CD14^high^, CD11b^low^CD14^low^, and neutrophil subsets in infected RMs (Figure 7B). In HTi RMs cells, parasite frequency was reduced in CD11b^low^CD14^low^ cells and neutrophils but persisted in monocytes/macrophages, with no significant difference compared to infected RMs (Figure 7B). In summary, we have clarified the identity of infected myeloid cell subsets that persist despite early administration of HePC.

## Discussion

In a model that closely resembles human physiology and anatomy, and well well-suited for studying human infection,^27^ we report the heterogeneity and reprogramming of splenic myeloid cell populations during *L. infantum* infection. Single-cell transcriptomic analysis reveals the persistence of these profiles in HTi RMs. This is associated with parasite persistence, despite the administration of HePC during the acute phase of infection. Indeed, *L. infantum* is disseminated in the spleen, PLNs, and MLNs, demonstrating its broad tissue distribution. Notably, the presence of parasites in the MLNs of infected RMs mirrors human disease, unlike the murine VL model, which lacks intestinal infection.^93^ We also observed that parasites in the PLNs were less sensitive to HePC-mediated killing during the early phase of treatment. Parasite burden was inversely correlated with cellular HePC concentration and varied by tissue. Therefore, HePC administration was insufficient to eradicate *L. infantum* in RMs, resulting in the establishment of parasite niches. This is a concern, as high relapse rates have been associated with insufficient drug exposure^94^ and the emergence of drug-resistant strains.^95^

The infection of monocytes and neutrophils by *Leishmania* is well documented.^96^ Parasites detected within splenic myeloid cells post-HTi may facilitate the dissemination to distal tissues. Indeed, splenic monocytes are mobilized in response to injury in inflamed tissues.^11^ Although PMNs are generally considered short-lived cells, our data intriguingly indicate that they can sustain infection within the spleen. This suggests that infected cells in tissues may persist longer than expected or become reinfected after HTi, potentially due to the release of amastigotes from splenic myeloid cells. Certain neutrophils have demonstrated plasticity in cancer, with prolonged survival due to environmental factors.^97^^,^^98^ Therefore, we cannot exclude the possibility that infected PMNs survive longer, as reported for myeloid cells,^99^^,^^100^ and may act as a Trojan horse for parasites, reducing treatment efficacy. This absence of full clearance may also be a mechanism by which drug-resistant parasites^95^ emerge due to drug selection. Therapeutic efficacy also depends on immune control of infection.^101^^,^^102^ In patients undergoing immunosuppressive therapy or co-infected with HIV, parasite relapse risks are increased,^103^^,^^104^ in which immunological factors predict relapse.^105^ Disrupted splenic architecture in *L. infantum*-infected RMs has been associated with reduced CXCL13 levels.^28^ The inability of HePC to restore CXCL13 levels, a marker of germinal center (GC) activity,^32^ suggests an alteration in lymphoid follicles. Consequently, future therapeutic strategies should focus on targeting infected cells that persist within these anatomical niches.

The presence of parasites in tissues may contribute to the reprogramming of myeloid cell polarization^106^^,^^107^ and DC maturation.^108^ Previous studies have reported mixed innate inflammatory and lymphoid signature expression associated with viscerotropic strains in mice,^25^^,^^109^ hamsters,^18^^,^^19^ dogs^17^ and humans.^23^^,^^110^ While treatment with AmBisome on chronically infected mice, eliminates parasites, gene expression was not completely restored to the baseline suggesting partial immune restoration.^111^ Similarly, altered transcriptional signatures have been observed in whole blood from patients with VL after amphotericin B or pentavalent antimonial treatment, despite parasite reduction.^23^^,^^110^ Using scRNA-seq combined with unsupervised analysis, we generated a cellular atlas of splenic myeloid cell populations, revealing the heterogeneity of these cells in *L. infantum-*infected RMs. Recently, a heterogeneity of myeloid cells was underlined in hepatic tissue from *L-infantum*-infected mice.^112^ Most importantly, we observed the persistence of altered immune profiles despite treatment during the acute phase. Therefore, assessing splenic myeloid cell profiles at single-cell resolution in RMs represents a significant advancement over bulk transcriptomic approaches.

Looking more in details about the altered profile of myeloid cell populations, we observed a shift in cDCs toward a cDC2 profile in *L. infantum*-infected RMs, which persisted despite HePC treatment. This shift is driven by the downregulation of genes associated with the cDC1 profile. A role of DC in visceral infection has long been recognized as crucial for infection control, notably by promoting Th1-dependent immunity.^113^ *In vitro* studies have demonstrated that *Leishmania-*induced reprogramming of DCs results in functional impairment^114^^,^^115^^,^^116^ and drives T cell polarization toward a non-protective phenotype.^117^^,^^118^^,^^119^ Herein, cDC2 exhibited a strong signature associated with TGF-β signaling (*NR4A1*, *NR4A2, RUNX2*, *RUNX3, FOXO1,* and *ATF3*) and fatty acid metabolism (*HADHA*, *ACOX1*, *HACD4,* and *MGLL*), which may impair antigen processing and presentation, reducing their ability to activate T cells.^120^ In parallel, we also observed an up-regulation of genes involved in the inflammasome pathway (*AIM2, CASP1, NOD2*, and *S100A4*). The role of the inflammasome in rodents remains controversial. It was reported that *L. amazonensis* induces a semi-mature DC phenotype with the down-regulation of inflammasome-related genes,^121^ while in macrophages, it controls parasite replication.^122^ In contrast, viscerotropic strains appear not to activate this platform, exploiting A20 and UCP2 proteins to inhibit inflammasome activation and to promote macrophage infection.^123^ In our study, *TNFAIP3* (A20) and *UCP2* were down-regulated in splenic cDC2 cells, suggesting potential inflammasome activation in this model.

Furthermore, our results highlighted inflamed monocytes following *Leishmania* infection, showing an enrichment of *NLRP1* and *NLRP3*, along with *MALT1*, a paracaspase involved in NF-κB signaling and implicated in noncanonical inflammasome activity.^124^ In addition to expressing genes related to cell migration, monocytes expressed higher levels of MCH molecules and ribosomal transcripts, indicating an activated state. A notable feature of these populations was the up-regulation of *IFNGR2*, a key activator of monocytes, essential for immune defense against *Leishmania* via the promotion of reactive oxygen species (ROS). Lipolysis could reflect cell membrane turnover related to cell migration^125^ and/or with cell fate switching from mitochondrial respiration to fatty acid oxidation due to the lower levels of *MT-ND3* and *MCL1*. These inflamed and activated splenic monocytes persist despite HePC treatment.

The profile of pDCs is also symptomatic of this immune dysregulation associated with *Leishmania* infection. The autophagic pathway is exacerbated in pDCs, which may result from lower expression of genes encoding organelles, including lysosomes and endosomes, leading to organelle dysfunction. Intriguingly, mtDNA gene levels are increased. This may reflect a dysregulation in the control of mitochondrial genome replication,^126^ as we found lower levels of genes regulating DNA/RNA replication (*TFUM*, *NAF1*, *NSD1*, *POLD1P3,* and *POLD4*), or the accumulation of mtDNA related to damaged and/or altered mitochondrial architecture,^46^ representing an autocrine damage-associated molecular pattern that activates pDCs. These findings could be important for the immune system, as pDCs contribute significantly to type I interferon (IFN) production, providing an early defense against pathogens.^127^ Schleicher et al. have previously reported that *in vitro L. infantum* induces IFN production by pDC.^128^ However, the role of pDCs in VL deserves further exploration, as type I IFNs have been shown to reduce adaptive immunity by suppressing CD4 T cell functions, potentially enhancing VL immunopathology.^24^

Finally, consistent with previous myeloid subsets, we highlighted a population of macrophages present only in *L. infantum* and HTi RMs. This population expresses an M2 profile associated with *CD163*, *CXCR4*, *IL4R*, and *ITGAM* (CD11b) transcripts, and higher levels of genes related to mitochondria, redox homeostasis, and proliferation (*CCND3*, *CCNG2*, *CDC5L,* and *PCNA*). This is relevant because, in addition to the description of M1/M2 signature in hamster,^18^ a splenic adherent proliferative macrophage was reported during VL.^129^ We found a higher level of *IL4R* transcripts in the infected RMs, which is consistent with a role for IL-4 in inducing macrophage proliferation.^130^ Our data also revealed the reprogramming of the M2 profile toward the M1 profile in HTi RMs, which may probably represent a transient profile. Indeed, this population displayed genes related to glycolysis, protein quality control, autophagy, ER, and ubiquitination.

The reprogramming of macrophages in HTi RMs is also sustained by the presence of higher levels of *APOE, ILF3,* and *IL2RA*, and lower levels of genes associated with M2 (*PLA2G7* and *MS4A6A*). In tingible body macrophages, we noticed higher levels of transcriptional factors that negatively regulate cellular functions (*KLF3*, *PAX5,* and *RCOR1*), while those associated with mitochondria and ER architectures were down-regulated (*OPA1* and *RTN4*), which may potentially compromise organelle^46^^,^^81^ and innate immune functions (*IFNGR1*, *CR2*, *CFP*, *MSR1,* and *PELI1*). Additionally, altered expression of *MT-ND3* was observed in both macrophage populations, which may have an impact on mitochondrial metabolism^131^ as reported in macrophages infected *in vitro* with *Leishmania*.^107^^,^^132^ We further observed lower levels of *GPX4*, a ferroptosis protein protecting against the accumulation of oxidized membrane lipids.^67^ Overall, our data reveal the extensive reprogramming of splenic myeloid cell populations during *Leishmania* infection.

In summary, our findings highlight the presence of *L. infantum* in various lymphoid sites despite treatment. The immune alterations observed, combined with early tissue dissemination, underscore the importance of targeting these niches to achieve a lasting VL cure. Single-cell transcriptomic analyses represent a breakthrough, revealing the heterogeneity and persistent innate immune alterations in the spleen of treated animals. This model closely mimics human physiology and anatomy, and it offers valuable insights into *Leishmania* niches and tissue immune alterations.

### Limitations of the study

This study has several limitations. Indeed, we focused on the early dissemination and niche establishment of *L. infantum*. Although the assessment of HePC in chronically infected RMs was not part of the present study, this opens an opportunity to explore whether the altered innate immune profile observed in early infection persists or changes over time, which could affect HePC efficacy. This study establishes a foundation for future research in this field.

It is important to note that this study focused only on females, and it cannot be ruled out that sex-related factors may contribute to exacerbated the disease in males.^133^

As previously mentioned, due to the lower abundance of myeloid cells in LNs, we analyzed only the transcriptomic profile of splenic cells. Given that *Leishmania* also persists in LNs, additional studies are warranted to assess the impact of parasite persistence on adaptive immune cells. A recent report on *L. donovani*-infected mice enabled a dual alignment of the *Leishmania* and rodent genomes,^92^ but this alignment of *L. infantum* with the macaque genome was inconclusive. Most reads were mapped to intergenic or intronic regions. This result highlights the necessity for methodological refinement, such as cell enrichment via cell sorting prior to scRNA analysis, to enhance sensitivity in detecting the *Leishmania* genome. In addition, the application of cell sorting to isolated populations can enable a more in-depth examination of potential niches.

A limitation of our scRNA approach was the analysis of PMNs, a population not captured after Ficoll-Paque density isolation but known to play a role in protozoal infections. Investigating different neutrophil subsets (N1 and N2) could provide insight into their contributions to infection control in VL.^134^^,^^135^

Our findings in RMs underscore the potential role of the gut in patients with VL by highlighting MLNs as key sites for parasite detection, a feature not typically observed in murine models.^136^ However, we cannot exclude the possibility that parasites persist in other tissues, such as the adipose tissue,^137^ a compartment not addressed here.

## Resource availability

### Lead contact

Requests for further information and resources should be directed to and will be fulfilled by the lead contact, Jérôme Estaquier (jerome.estaquier@crchudequebec.ulaval.ca).

### Materials availability

This study did not generate new unique reagents.

### Data and code availability

• Single cell RNA-seq data have been deposited at the GEO depository as GSE307619.
• This article does not report the original code.
• Any additional information required to reanalyze the data reported in this article is available from the lead contact upon request.

## Acknowledgments

We acknowledge Stéphanie Blanc and Hugues Contamin (Cynbiose, Marcy l’Etoile, France) for the non-human primate housing and their support. We also thank Stéphanie Dupuy from BioMedTech Facilities (INSERM US36, CNRS UMS2009, Université Paris Cité, France) for the cytometry platform. This work was supported by the European Community’s Seventh Framework Programme under grant agreement No.602773 (Project KINDRED) and grant agreement N°603240 (Project NMTryp), and Infect-ERA ERA-NET (Project InLeish) to JE. APC, ACs, and JE are members of the COST Action CA21111: One Health Drugs against Parasitic Vector-Borne Diseases in Europe and Beyond (OneHealthDrugs). This work has also been funded by National funds, through the 10.13039/501100019370Foundation for Science and Technology (FCT) - project UIDB/50026/2020 (https://doi.org/10.54499/UIDB/50026/2020), UIDP/50026/2020 (https://doi.org/10.54499/UIDP/50026/2020), and LA/P/0050/2020 (https://doi.org/10.54499/LA/P/0050/2020). RD is supported by FCT contract 10.54499/2020.00185.CEECIND/CP1600/CT0004. JE also thanks the Canada Research Chair program for financial assistance. SA was supported by a post-doctoral fellowship granted by Fédération pour la Recherche Médicale (FRM: SPF20160936115) and Infect-Era (ANR-16-IFEC-0002). MP by a doctoral fellowship granted by Infect-Era (ANR-16-IFEC-0002) and SB by a fellowship support from Formation Desjardins pour la Recherche et l’Innovation (CHU de Québec). VR by a post-doctoral fellowship granted by KINDRED.

## Author contributions

Investigation and formal analysis, M.P., S.B., V.R., Y.F., C.B., C.S., J.C., C.J.B., G.R., O.Z.A., and S.A.; supervision, A.D., A.P., S.A., and J.E.; funding acquisition, M.P.C., R.S., J.M.D., and J.E.; writing – review and editing, M.P., M.P.C., R.S., A.C.S., J.M.D., S.A., and J.E.

## Declaration of interests

The authors declare no competing interests.

## STAR★Methods

### Key resources table

**Table:** 

REAGENT or RESOURCE	SOURCE	IDENTIFIER
**Antibodies**		

Anti-Non-Human Primate CD3-V500	BD Biosciences	Cat#560770; RRID: AB_1937322
Anti-human CD11b-PE	BD Biosciences	Cat#555388; RRID: AB_395789
Anti-human CD20-APC-H7	BD Biosciences	Cat#560734; RRID: AB_1727449
Anti-human CD14-FITC	Miltenyi Biotec	Cat#130-080-701; RRID: AB_244303
Anti-human HLA-DR-PerCP	Miltenyi Biotec	Cat#130-095-291; RRID: AB_10839556
Anti-Non-Human Primate CD45-PE	BD Biosciences	Cat#552833. RRID: AB_394483

**Biological samples**		

Blood, peripheral and mesenteric lymph nodes, spleen and bone marrow are from *Macaca Mulatta*	Cynbiose SAS, VetAgro-Sup	N/A

**Chemicals, peptides, and recombinant proteins**		

Miltefosine	MedChemExpress	Cat#HY-13685
Acetonitrile RS - Pour UHPLC-MS	Carlo Erba Reagents	Cat#412042
alamarBlue™ Cell Viability Reagent	Invitrogen	Cat#DAL1100

**Critical commercial assays**		

QIAamp DNA Mini Kit	Qiagen	Cat#51306
TaqManTM Universal Master Mix II, no UNG	Applied Biosystems	Cat#4440049
SensiFASTTM SYBR® Hi-ROX Kit	Bioline	Cat#BIO-92020
Human IL-1ra/IL-1F3 Quantikine ELISA Kit	Biotechne	Cat#DRA00B
Human CXCL13/BLC/BCA-1 ELISA Kit	Biotechne	Cat#DCX130
LEGENDplex™ NHP Chemokine/Cytokine Panel	Biolegend	Cat#740330
Calcein AM	ThermoFisher Scientific	Cat#C1430
DRAQ7™	BD Pharmingen	Cat#564904
BD Rhapsody™ Enhanced Cartridge Reagent Kit	BD Biosciences	Cat#664887
BD Rhapsody™ Cartridge Kit	BD Biosciences	Cat#633733
BD Rhapsody™ cDNA Kit	BD Biosciences	Cat#633773
BD® Flex Sample Multiplexing Kit A Tag 1-6	BD Biosciences	Cat#633849
Qubit™ dsDNA Quantification Assay Kit	Invitrogen	Cat#Q32851

**Deposited data**		

Single-cell RNA-seq data	This paper	GSE307619 https://can01.safelinks.protection.outlook.com/?url=https%3A%2F%2Fwww.ncbi.nlm.nih.gov%2Fgeo%2Fquery%2Facc.cgi%3Facc%3DGSE307619&data=05%7C02%7CJerome.Estaquier%40crchudequebec.ulaval.ca%7C3ad36450c6ee440af17408de0d80252f%7Ccb779487921b4201825c1ed13e6f1b40%7C0%7C0%7C638963041195577790%7CUnknown%7CTWFpbGZsb3d8eyJFbXB0eU1hcGkiOnRydWUsIlYiOiIwLjAuMDAwMCIsIlAiOiJXaW4zMiIsIkFOIjoiTWFpbCIsIldUIjoyfQ%3D%3D%7C0%7C%7C%7C&sdata=6Shl7qCjLtYocoh3K6qMXyWXQFktHi39DfhwIbCDV9w%3D&reserved=0 Token can be obtained on request to Jerome Estaquier

**Experimental models: Cell lines**		

*Leishmania infantum* (MHOM/MA/67/ITMAP-263)	Centre de Ressources Biologiques des *Leishmania* (CRBL)	https://crb-leish.edu.umontpellier.fr/

**Experimental models: Organisms/strains**		

*Macaca Mulatta*	Cynbiose SAS, VetAgro-Sup	N/A

**Oligonucleotides**		

Kinetoplastid FP:5’-CTTTTCTGG TCCTCCGGGTAGG-3’	This paper	N/A
Kinetoplastid RP: 5’-CCACCCGG CCCTATTTTACACCAA-3’	This paper	N/A
Kinetoplastid Probe: 5′FAM-TTTTCGCAGA ACGCCCCTACCCGC-3′TAMRA	This paper	N/A
Albumin FP: 5’-CCATTGGTG AGACCAGAGGT-3’	This paper	N/A
Albumin RP: 5’-GAGGCAGGC AGCTTTATCAG-3’	This paper	N/A

**Software and algorithms**		

GraphPad Prism (v8.0.1)	GraphPad Software	https://www.graphpad.com/
FlowJo (v10.10)	BD Biosciences	https://www.flowjo.com/
Seven Bridges BD Rhapsody Sequence Analysis Pipeline	SevenBridges	https://www.bdbiosciences.com/en-us/products/software/rhapsody-sequence-analysis-pipeline
R (v4.4.0)	R Core Team, 2024	https://www.R-project.org
Seurat package (v5.2.1)	Hao et al.^138^	https://satijalab.org/seurat/
ggplot2 (v3.5.2)	Wickham et al.^139^	https://link.springer.com/book/10.1007/978-3-319-24277-4
Dplyr (v1.1.4)	Wickham et al.^139^	https://dplyr.tidyverse.org.
Tidyr (v1.3.1)	Wickham et al.^139^	https://tidyr.tidyverse.org.
Openxlsx (v4.2.8)	Schauberger et al.^140^	https://ycphs.github.io/openxlsx/index.html
EnhancedVolcano package (v1.22.0)	Blighe et al.^141^	https://bioconductor.org/packages/release/bioc/html/EnhancedVolcano.html
ToppGene Suite	Chen et al.^106^	https://toppgene.cchmc.org/enrichment.jsp
g:Profiler	Raudvere et al.^142^	https://biit.cs.ut.ee/gprofiler/gost
Gene Ontology Enrichment Analysis	Ashburner et al.^143^	https://geneontology.org

**Other**		

Genome of *Macaca mulatta*	Gokey et al.^144^	GCA_003339765.3
Genome of *Leishmania infantum*	Gonzalez-de la Fuente et al.^145^	GCA_900500625.2

### Experimental model and study participant details

#### Animals, parasites, and infections

Ten female rhesus macaques (*Macaca mulatta*) of Chinese origin were used in this study. Animals, aged 40 months and weighing 4.5 ± 0.3 kg each, were seronegative for STLV-1 (Simian T Leukemia Virus type-1), SRV-1 (type D retrovirus), Herpes-B viruses, and SIVmac. All animals were housed in compliance with French regulations for animal care and use (Cynbiose SAS, VetAgro-Sup, Marcy l’Étoile, France) and animal experiments were reviewed by the Animal Welfare Body of Cynbiose and the Ethics Committee of VetAgro-Sup approved under the number 4305-2016020215429763. All experiments were conducted in accordance with the European Directive 2010/63/UE as published in the French Official Journal of February 7th, 2013. Animals were inoculated intravenously via the saphenous vein with 2x10^7^ stationary-phase *L. infantum* promastigotes (clone MHOM/MA/67/ITMAP-263) per kg of body weight as previously described.^28^ At day one post-infection RMs were treated for 21 days by nasogastric gavage with 5 mg/kg of HePC (Sigma-Aldrich). RMs were sacrificed at three time points (weeks 5, 9 and 12 p.i., corresponding to weeks 2, 4 and 9 post-HTi). Tissues were collected after necropsy, including axillary and inguinal peripheral LNs (PLNs), mesenteric LNs (MLNs), that drain the small and the large intestine, spleen, bone marrow (BM) aspirates, and peripheral blood. Tissues were immediately mechanically processed. Indeed, they were not digested with collagenase or other proteases to limit negative effects on the expression of cell surface markers, particularly for CD14 expression that can be shed.^146^ A hemogram was performed by Biovelys (Vet Agro Sup, Lyon).

### Method details

#### Parasite quantification

DNA was extracted from cell pellets of blood or tissues using the QIAamp DNA Mini Kit (Qiagen) according to the manufacturer’s protocol. A TaqMan-based qPCR assay was used to detect and quantify *L. infantum* kinetoplastid DNA minicircles as described previously.^28^ Briefly, mixtures were composed of TaqMan Universal Master mix II, no UNG (2X, Applied Biosystems), 400 nM of forward and reverse primers and 250 nM of TaqMan probe. Thermocycling settings consisted of one hold of 15 min at 95°C followed by a two-step temperature (95°C for 15 s and 60°C for 60 s) over 40 cycles in an ABI Prism 7900 HT (Applied Biosystems). A standard curve was established, and sample normalization was performed by quantifying the macaque albumin host gene. Thus, parasite load was expressed as kinetoplastid copy number/10^6^ nucleated cells.

#### Quantification of cytokines/chemokines by ELISA

ELISA was used to quantify IL-1Ra (Human IL-1ra/IL-1F3, R&D Systems) and CXCL13 (Human CXCL13/BLC/BCA-1, R&D Systems), while CXCL10/IP-10 was quantified by flow cytometry using a LEGENDplex assay (Biolegend) and standards were used for quantification according to the manufacturer’s instructions.

#### Parasite rescue assay

Parasites were quantified in the spleen and peripheral LN (PLN) using culture microtitration.^39^ Briefly, the cells were resuspended in Schneider’s drosophila medium (Gibco) supplemented with 20% heat-inactivated FCS (Gibco), penicillin/streptomycin (100 U/mL, Life technologies), glutamine (2 mM, Life technologies) and sodium pyruvate (1 mM, Life technologies). Serial dilutions were performed in a 96-well plate ranging from 1x10^6^ to 5 cells. The plates were sealed hermetically and incubated at 26°C for 21 days to allow promastigote conversion and multiplication. After that, the wells were incubated with Alamar blue reagent (ThermoFisher) at 37°C, and after overnight incubation, fluorescence was monitored (λex=560 nm and λem=590 nm) on an EnSpire plate reader (Perkin Emer).^147^ Parasite density was calculated through a standard curve ranging from 1x10^7^ to 1 *L. infantum* promastigotes.

#### HePC quantification

Pharmacokinetics of HePC were performed in the sera and from the pellet of PLN, MLN and splenic cells stored at -80°C. Proteins from frozen pellets were precipitated with acetonitrile and were analyzed, along with the sera, the same day to limit variation. Ultra-performance liquid chromatography (UPLC) system (Waters ACQUITY) with a 2.1x50-mm, 1.7-m ACQUITY UPLC BEH RP18 shield column coupled to a Waters Qua9ro Premier TQ mass spectrometer was used and operated in positive ion electrospray and multiple reaction monitoring mode (MRM). Miltefosine was used as a calibration standard and was run in parallel the same day to limit inter-day variability, mobile phase composition, column temperature and sample stability.^148^

#### Cell sorting and immunophenotyping

Cells from the spleen were stained with a cocktail of antibodies, including anti-CD3 (clone SP34-2), anti-CD11b (clone ICRF44), and anti-CD20 (clone 2H7) from BD biosciences and anti-CD14 (clone TÜK-4) and anti-HLA-DR (clone AC122) from Miltenyi Biotec. The cells were then fixed with paraformaldehyde and sorted (FACS ARIA III cytometer, BD Biosciences) for DNA parasite quantification by qPCR. The purity of each cell type was higher than 97%.

#### Single-cell library preparation and sequencing

Spleen cells were isolated using a Ficoll-Paque density gradient solution (Sigma). Cell suspensions were labeled with a primary CD45 antibody. The BD™ Flex Single-Cell Multiplexing Kit was used to combine and load four samples onto a single BD Rhapsody cartridge according to manufacturer’s guide. Thus, an average of 40 000 pooled cells were loaded onto the cartridge. Single cell isolation was followed by cell lysis and poly-adenylated mRNA molecules with barcoded and captured with magnetic beads according to the manufacturer's instructions. Thus, a unique molecular identifier (UMIs) was added to the cDNA molecules. Quantity and quality of the sequencing libraries were analyzed with the Qubit dsDNA HS assay kit (Invitrogen) and the 4200 TapeStation system (Agilent). Libraries were sequenced on the Novaseq 6000 system (Illumina) targeting a sequencing depth of 50.000 reads/cell and 600 reads/cell/Sample Tag for sample tag library. To conduct single-cell analysis, the genomic sequence for *Macaca mulatta* was obtained from the Ensemble database (accession number GCA_003339765.3). The raw sequencing reads were processed using the BD Rhapsody™ analysis pipeline, which included read quality filtering, annotation, putative cell identification, and the generation of single-cell gene expression matrices. Two technical replicates were generated and integrated for downstream analysis. The integrated data were analyzed using the Seurat package (v5.2.1). Cells were first log-normalized using the NormalizeData function (method = “LogNormalize”, scale.factor = 10,000), ensuring that gene expression values were scaled proportionally across cells. Following normalization, data were scaled using ScaleData, and a dimensionality reduction was performed via principal component analysis on highly variable genes. The top 20 principal components were used to construct a shared nearest neighbor graph (FindNeighbors), and cell clustering was performed using FindClusters, with resolutions ranging from 0.3 to 0.5 depending on the analysis. Genes associated with non-annotated loci (prefix ENSMMUG) were filtered out prior to downstream analyses.

#### Single-cell RNA-seq visualization

Cellular relationships were visualized using Uniform Manifold Approximation and Projection (UMAP), with parameters set as follows: dims = 1:20, n.neighbors = 50, min.dist = 0.3, and spread = 1. Unsupervised identification of cluster markers was performed using the FindAllMarkers function, restricted to positively differentially expressed genes (only.pos = TRUE), with thresholds set at min.pct = 0.35 and a log fold-change cutoff (logfc.threshold = 1). A post-hoc filtering step was applied to retain only markers with a difference of at least 25% compared to other clusters (pct.1 - pct.2 ≥ 0.25). For each retained gene, p-values were corrected for multiple testing using the Benjamini-Hochberg method to control the false discovery rate (FDR). The top 10 marker genes were selected based on adjusted p-values and average log2 fold-change and were visualized using the DoHeatmap function. Supervised heatmaps were generated by selecting predefined lists of genes of interest, which were visualized using the DotPlot function, allowing for the comparative expression analysis across clusters.

#### Cluster-specific differential expression analysis

Each cluster was individually isolated, and differential gene expression analyses were performed between the three experimental conditions (L. infantum-infected, treated and naïve RMs) using the FindMarkers function. For each comparison, differentially expressed genes were selected based on a threshold of p-value < 0.05, |log2 fold change| > 1, and a mean raw expression greater than 1 in at least one of the two compared groups. Results were visualized using volcano plots generated via the EnhancedVolcano package (v1.22.0). Dots were colored according to statistical significance and fold-change thresholds, with genes meeting significance criteria highlighted.

#### Functional enrichment analysis

To functionally characterize the differentially expressed genes, enrichment analyses were performed using ToppFun (ToppGene Suite),^149^ g:Profiler, and Gene Ontology (GO) Biological Processes annotations.^143^ Pathway enrichment was conducted separately for upregulated and downregulated gene sets. To gain a deeper understanding of the signaling pathways, we employed an iterative approach. After identifying the first set of genes, these were excluded from further analysis, and the remaining genes were reprocessed. This strategy minimizes the redundancy of pathways associated with only few genes, offering a more accurate view of the modulated pathways.

### Quantification and statistical analysis

Statistics were performed with the GraphPad Prism 8 software. Data are presented as means ± SEM. Statistical differences were assessed using the Mann-Whitney U test (∗, P < 0.05; ∗∗, P < 0.01; ∗∗∗, P < 0.001) and are indicated in the figure legends.

## Supplemental information

Document S1. Figures S1–S8

Table S1. Genes characterizing Cluster 0, Genes characterizing Cluster 1, Genes characterizing Cluster 3, Genes characterizing Cluster 4, Genes characterizing Cluster 5, Genes characterizing Cluster 6, Genes characterizing Cluster 7

Table S2. DEG between *L. infantum* and naive RM, Cluster 0

Table S3. DEG between HTi and naive RM, Cluster 0

Table S4. DEG between *L. infantum* and naive RM, Cluster 1

Table S5. DEG between HTi and naive RM, Cluster 1

Table S6. DEG between *L. infantum* and naive RM, Cluster 3

Table S7. DEG between HTi and naive RM, Cluster 3

Table S8. DEG between *L. infantum* and naive RM, Cluster 4

Table S9. DEG between HTi and naive RM, Cluster 4

Table S10. DEG between *L. infantum* and naive RM, Cluster 5

Table S11. DEG between HTi and naive RM, Cluster 5

Table S12. DEG between HTi and naive RM, Cluster 6

Table S13. Number of Leishmania reads (LINF) Two runs are indicated (run 1 and run 2) in naive, infected and HTi RMs (nd: not detected).
