# Supplementary material for: Persistent myeloid cell reprogramming despite miltefosine treatment in leishmania-infected macaques
Source: iScience. 2025 Nov 3;28(12):113900. doi: 10.1016/j.isci.2025.113900 (PMC12663736; doi:10.1016/j.isci.2025.113900)
Supplement: Document S1. Figures S1–S8 [file mmc1.pdf]

iScience, Volume *28*

Supplemental information

Persistent myeloid cell reprogramming despite miltefosine treatment in leishmania-infected macaques

Morgane Picard, Steven Boutrais, Vasco Rodrigues, Yasmina Fortier, Chloé Borde, Calaiselvy Soundaramourty, Julien Clain, Charles Joly-Beauparlant, Gina Racine, Ouafa Zghidi-Abouzid, Arnaud Droit, Alain Pruvost, Maria Paola Costi, Ricardo Silvestre, Anabela Cordeiro da Silva, Jane MacDougall, Sónia André, and Jérôme Estaquier

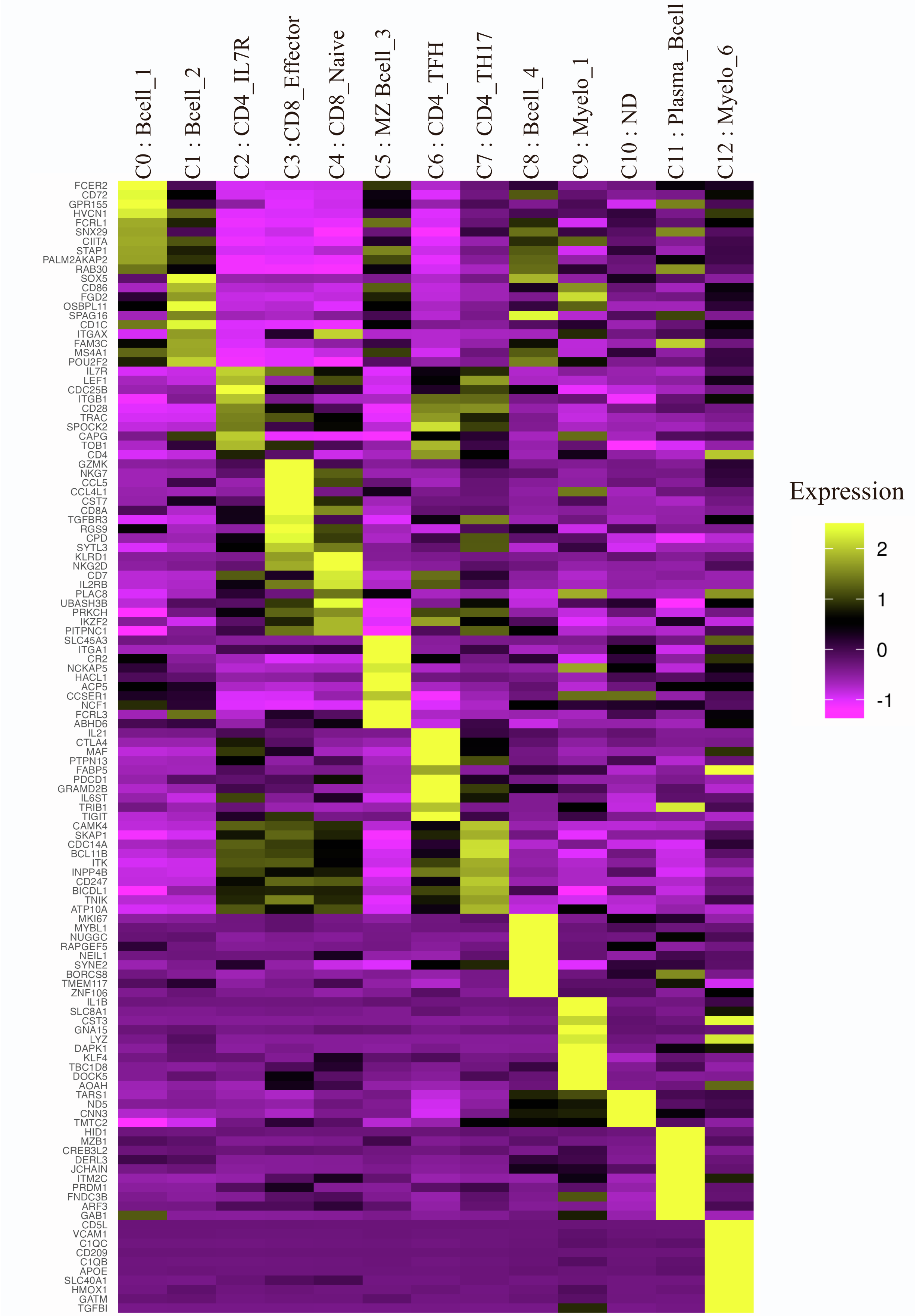

Supplemental Figure 1: Heatmap of differentially expressed genes associated with scRNA analysis.

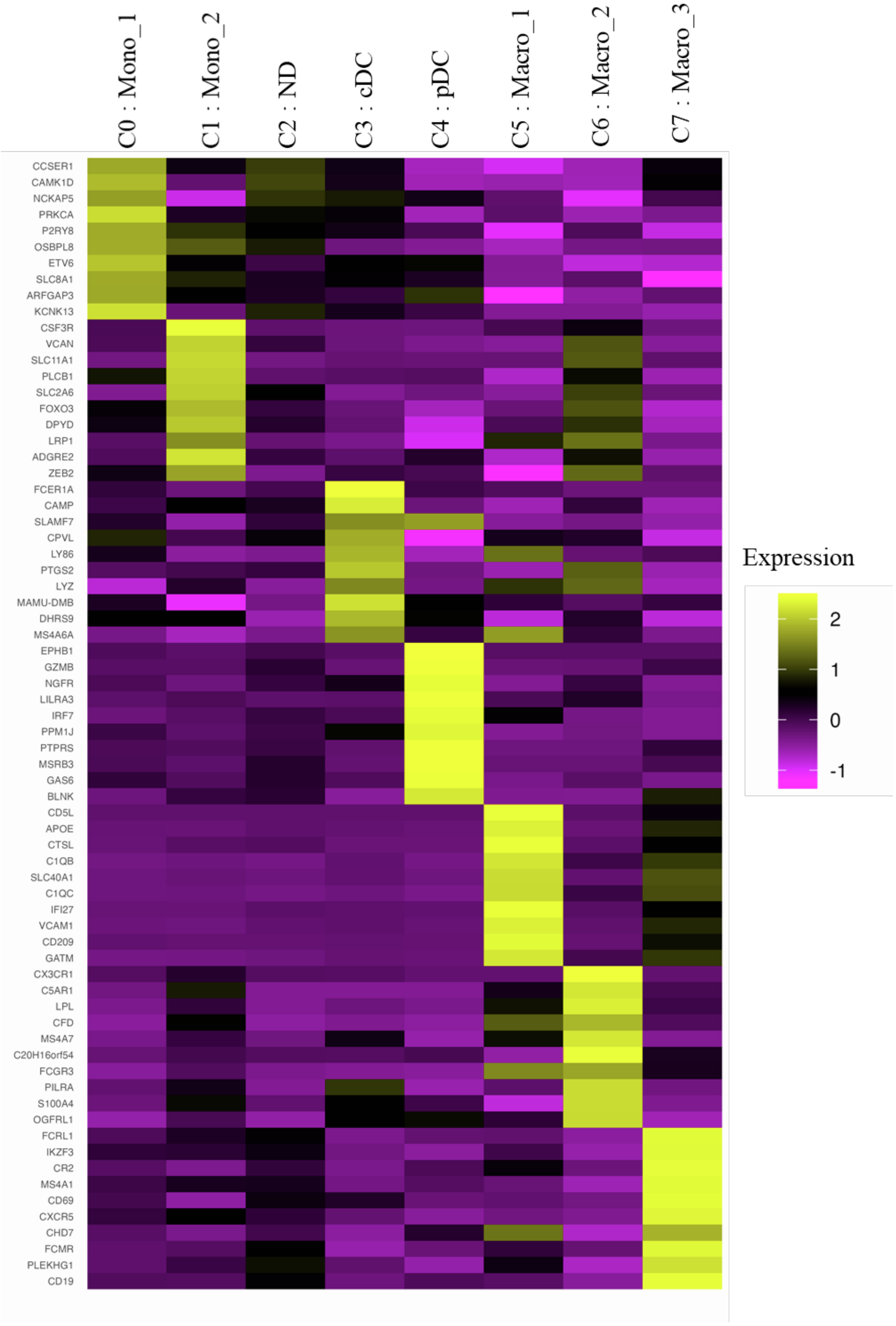

Supplemental Figure 2: Heatmap of differentially expressed genes associated with clusters 9 and 12.

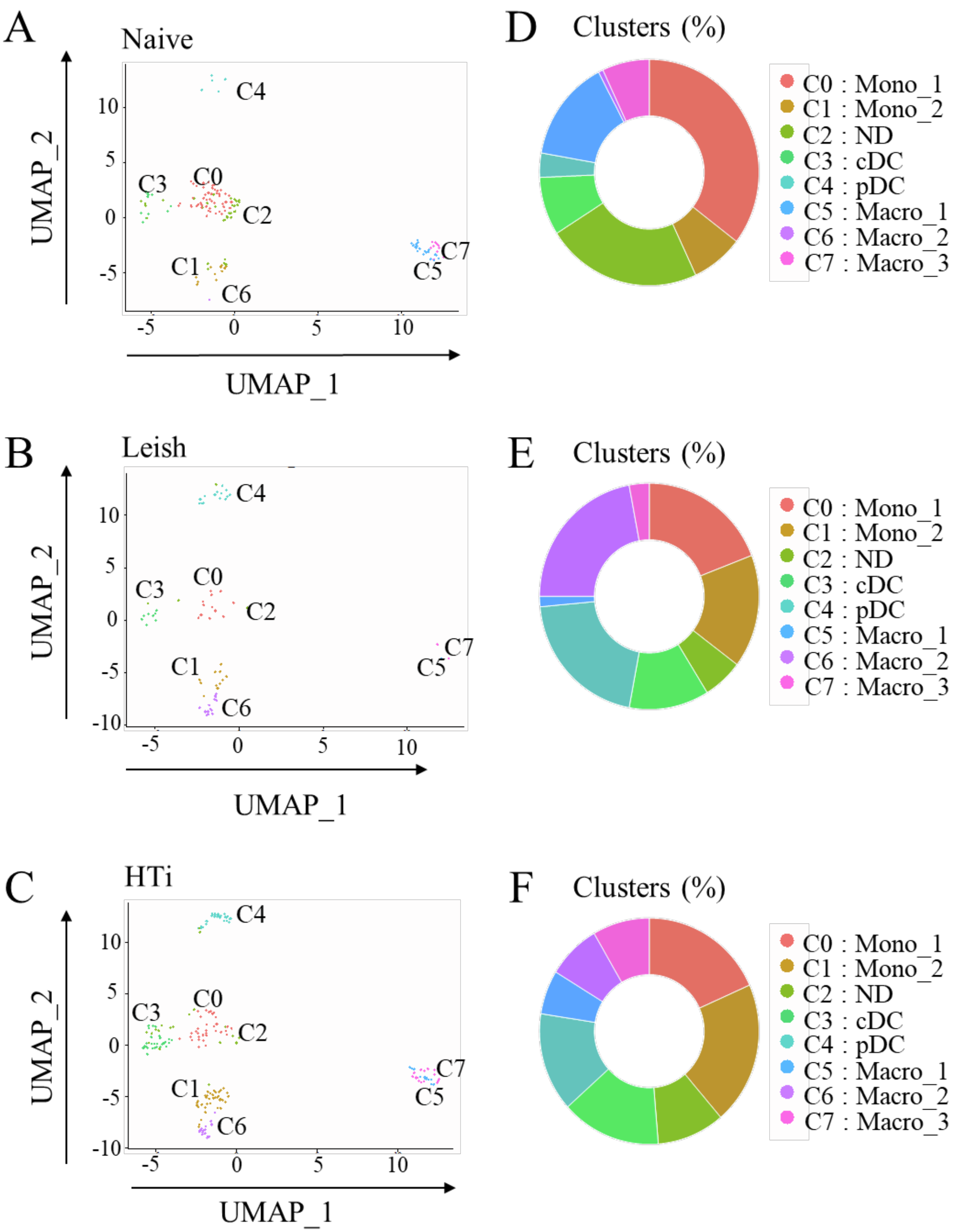

Supplemental Figure 3: **Transcriptomic signature of myeloid cells according to specific RMs.** (A-C) UMAP projection of myeloid cells from each condition. (A) Naïve RMs, (B) *L. infantum*-infected RMs, (C) and HTi RMs at weeks 2 and 4 post-HePC interruption. (D-F) Diagrams show the proportions of myeloid cell subpopulations for each condition. Each color represents one cluster.

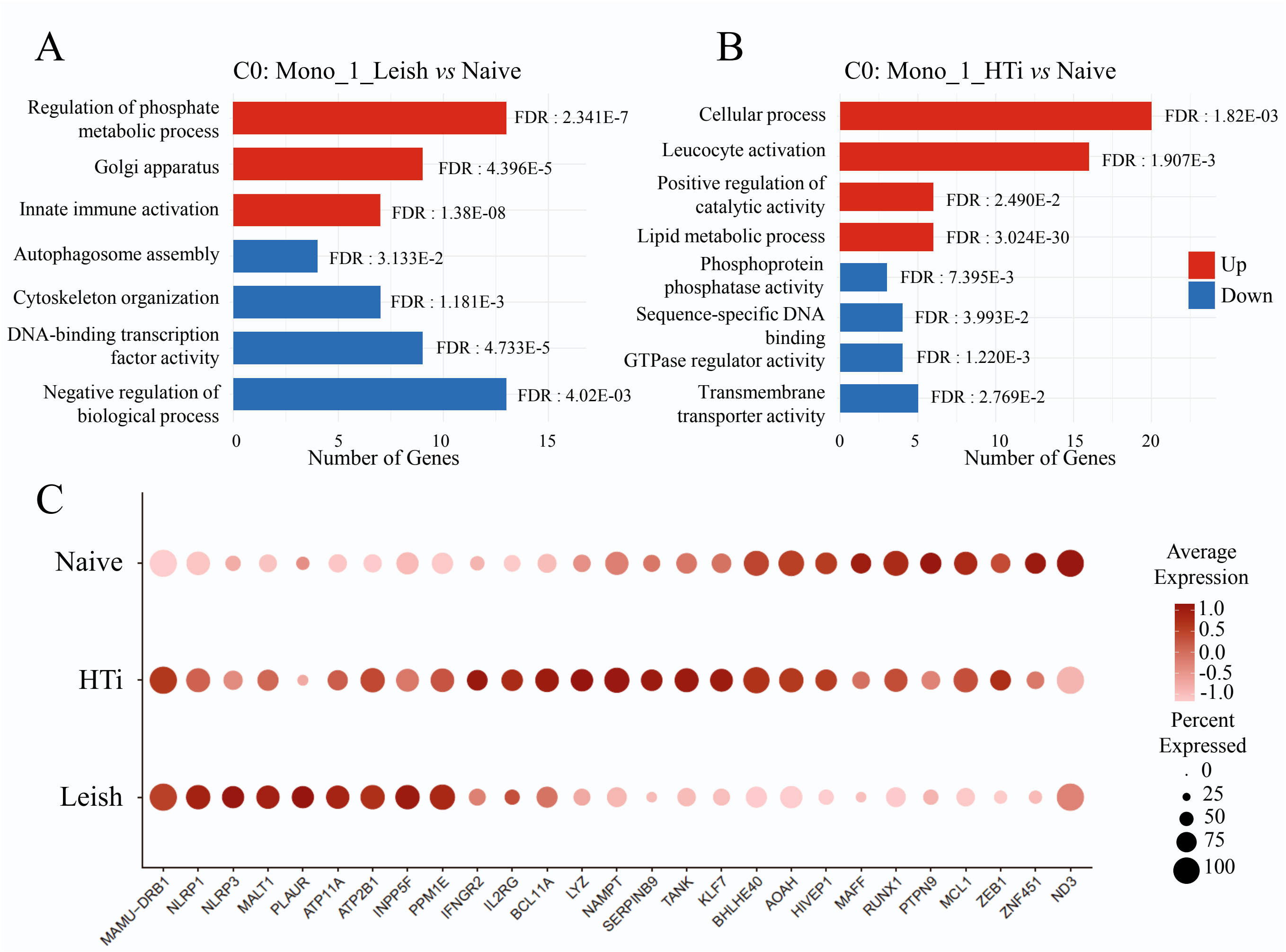

Supplemental Figure 4: Pathway enrichment of cluster 0 (Mono_1). A) *L. infantum*-infected *versus* naïve RMs and B) HTi *versus* naïve RMs. C) Heatmap highlighting genes of interest. The size of the dot represents the percentage of cells expressing the selected gene in each cluster and its average.

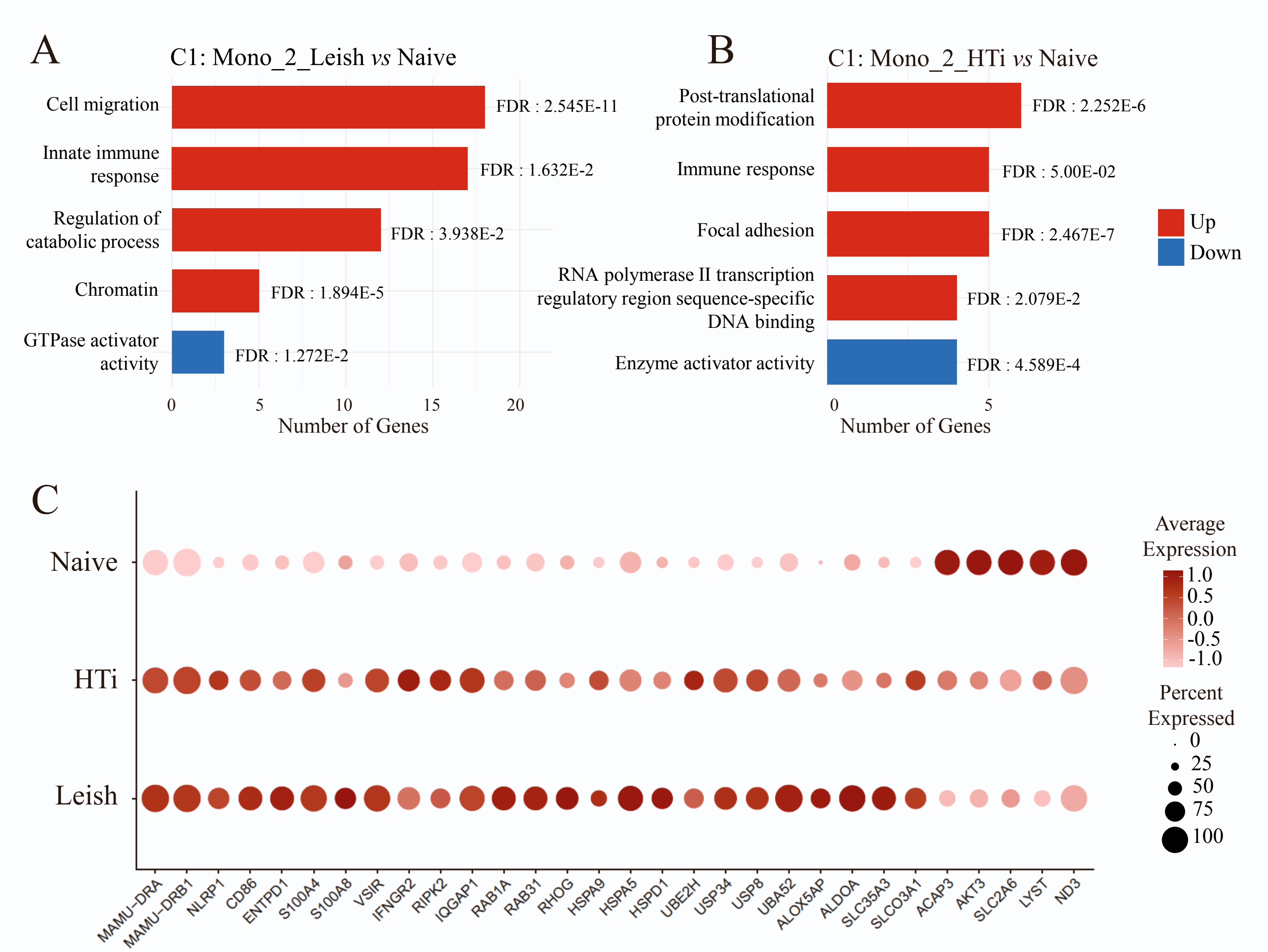

Supplemental Figure 5: Pathway enrichment of cluster 1 (Mono_2). A) *L. infantum*-infected *versus* naïve RMs and B) HTi *versus* naïve RMs. C) Heatmap highlighting genes of interest. The size of the dot represents the percentage of cells expressing the selected gene in each cluster and its average.

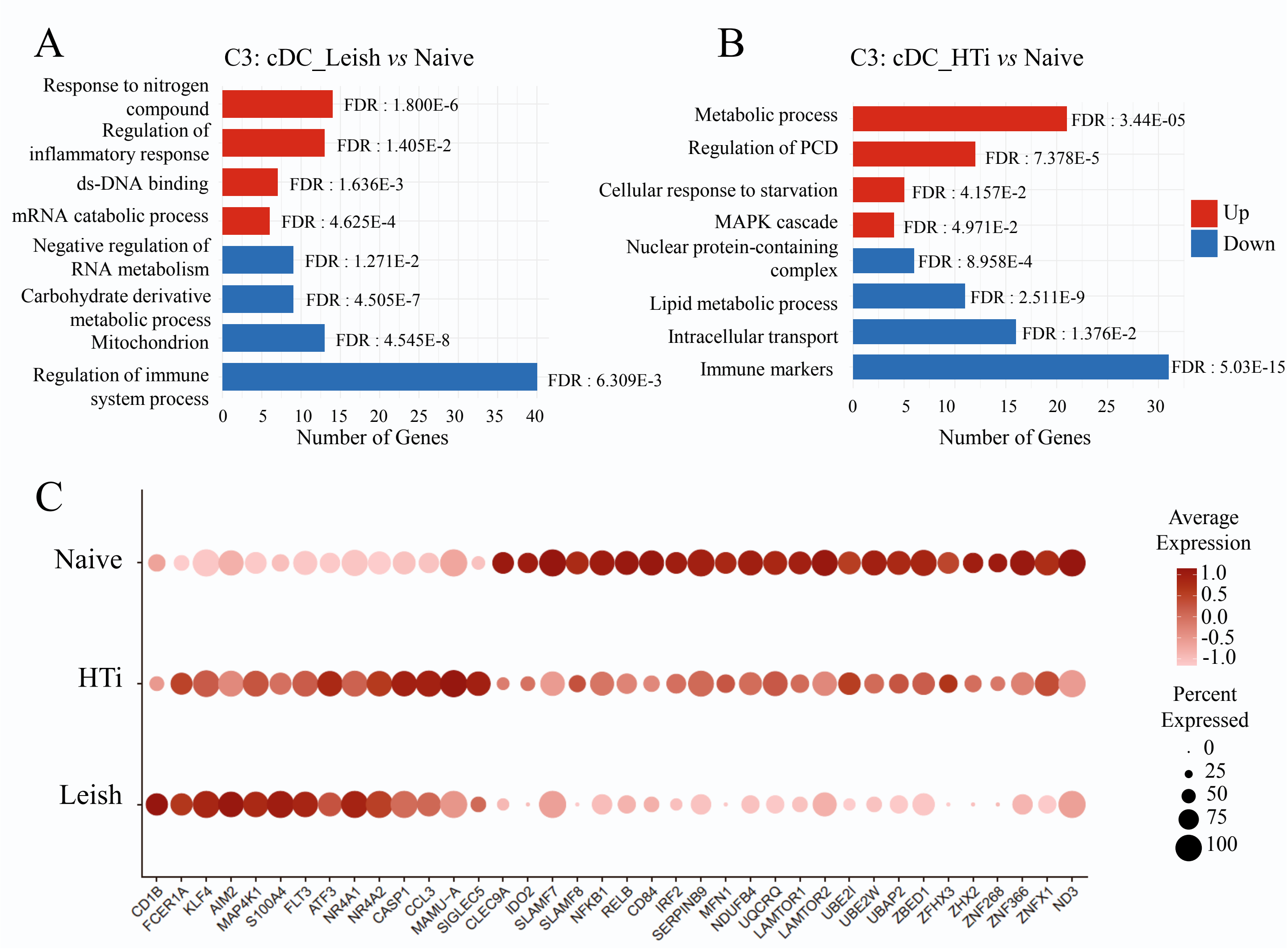

Supplemental Figure 6: Pathway enrichment of cluster 3 (cDC). A) *L. infantum*-infected *versus* naïve RMs and B) HTi *versus* naïve RMs. C) Heatmap highlighting genes of interest. The size of the dot represents the percentage of cells expressing the selected gene in each cluster and its average.

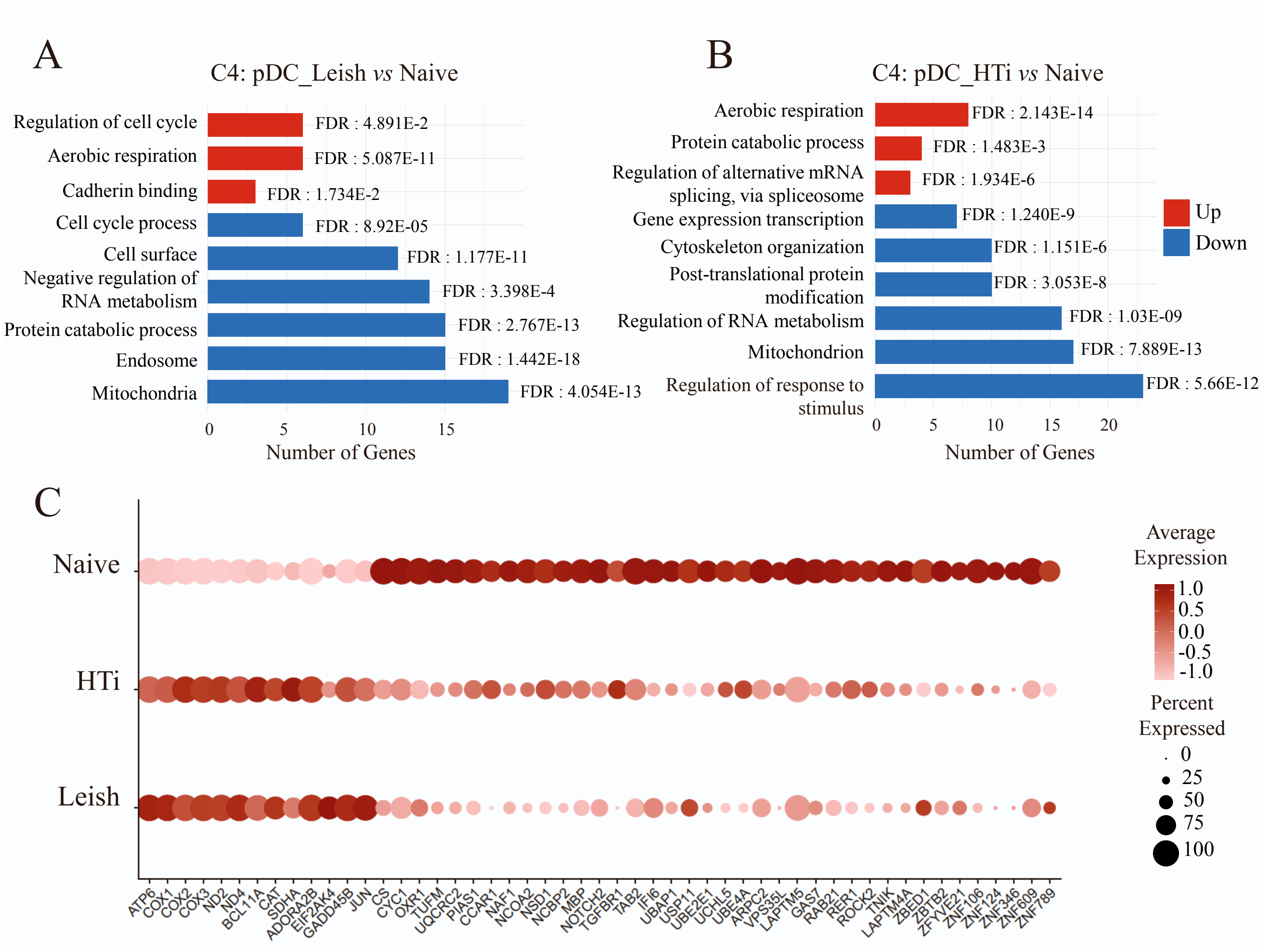

Supplemental Figure 7: Pathway enrichment of cluster 4 (pDC). A) *L. infantum*-infected *versus* naïve RMs and B) HTi *versus* naïve RMs. C) Heatmap highlighting genes of interest. The size of the dot represents the percentage of cells expressing the selected gene in each cluster and its average.

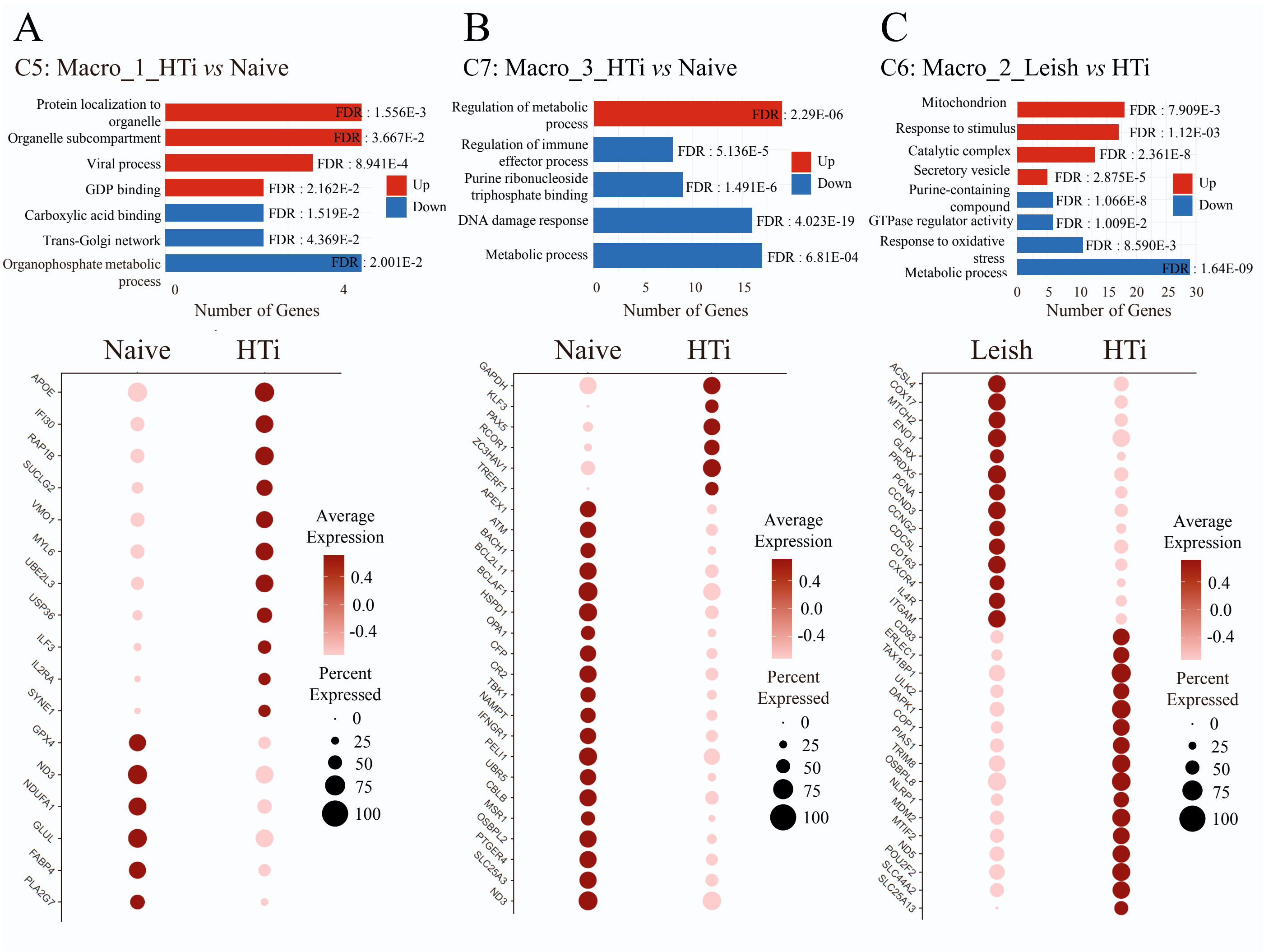

Supplemental Figure 8: Pathway enrichment of cluster 5, 6 and 7 (Macro). A) Hti *versus* naïve RMs (cluster 5, Macro_1), B) HTi *versus* naïve RMs. (cluster 7, Macro_3) and C) *L. infantum*-infected *versus* HTi RMs (cluster 6, Macro_2). Heatmaps highlighting genes of interest are represented by the percentage of cells expressing the selected gene (dot size) and its average in each cluster.
